# Effects of different prehabilitation programs on the major abdominal surgery population: a systematic review and network meta-analysis

**DOI:** 10.3389/fmed.2025.1673338

**Published:** 2026-01-14

**Authors:** Yue Sun, Wenchao Mao, Yaoyuan Li, Yan Sun, Kaixuan Li, Zheng Wang, Dongpo Zhang, Hengxin Bai, Han Xia, Xiaoli Zhang, Han Zhao, Qingshuang Wei, Quanda Liu, Baohui Jia

**Affiliations:** 1 Department of Rehabilitation, Guang’anmen Hospital, China Academy of Chinese Medical Sciences, Beijing, China; 2 Department of General Surgery, Guang’anmen Hospital, China Academy of Chinese Medical Sciences, Beijing, China

**Keywords:** intervention strategy, major abdominal surgery, network meta-analysis, prehabilitation program, review

## Abstract

**Background:**

Patients undergoing major abdominal surgery experience high rates of postoperative complications, mortality and healthcare utilization. Prehabilitation is intervention to enhance functional capacity before surgery, aimed at improving the patient’s tolerance to upcoming physiologic stress. We performed a network meta-analysis (NMA) to evaluate the relative effects of different prehabilitation programs on improving fitness and surgical outcomes in patients with the major abdominal surgery.

**Methods:**

We searched PubMed, Embase, Web of Science, Google Scholar, and Cochrane from inception to June 2025. Randomized controlled trials (RCTs) investigating prehabilitation programs for major abdominal surgery related outcomes of peak volume of oxygen uptake (VO_2peak_), 6-min walk test (6MWT), length of stay (LOS), and postoperative complications were included. The frequentist random-effect NMA method was used to pool the results.

**Results:**

We included 31 studies with 2,467 participants for meta-analysis. High-intensity interval training (HIIT) was the most effective intervention in improving VO_2peak_ (SUCRA = 73.9%, MD = 2.29, 95% CI: 0.52–4.06), and aerobic exercise was probably the best intervention for increasing 6-min walking distance (SUCRA = 98.0%, MD = 71.67, 95% CI: 17.44–125.90) that achieved the minimal clinical difference. Multimodal interventions may be more advantageous in reducing LOS (SUCRA = 74.7%, MD = –1.50, 95% CI: –3.02 to –0.02), and HIIT was the most promising prehabilitation program in reducing postoperative complications (SUCRA = 98.4%, OR = 0.03, 95% CI: 0.00–0.51).

**Conclusion:**

Based on limited quality and direct evidence, our preliminary findings showed that HIIT tended to be most effective in enhancing VO_2peak_ and reducing postoperative complications in patients undergoing major abdominal surgery. Aerobic exercises were more effective in increasing 6-min walking distance, and multimodal interventions were more advantageous in reducing LOS. Treatment strategies should be based on the patient’s condition and comprehensively determined through real-time evaluation and monitoring.

**Systematic review registration:**

https://www.crd.york.ac.uk/PROSPERO/, identifer CRD42024545664.

## 1 Introduction

Patients undergoing major abdominal surgery are primarily individuals suffering from abdominal tumors, with the majority being over 60 years old (1, 2). These patients frequently exhibit impaired cardiopulmonary function, considerable loss of strength, weakness, sarcopenia, and malnutrition (3). These characteristics are linked to poor patient outcomes and have an impact on daily activity performance (1, 4, 5), which contributed considerably to the postoperative length of stay (LOS) and increased hospital costs (6).

Prehabilitation programs aim to reduce risk of postoperative complication in frail and less fit patients by emphasizing preoperative training to improve their functional psychophysiological reserve (7, 8). This technique may benefit patients undergoing abdominal cancer surgery for gastrointestinal, urological, gynecological, hepatobiliary, and pancreatic malignancies by improving their post-operative recovery and short-term outcomes (8). For example, it is thought that this type of prehabilitation will result in a faster recovery of physical functioning, less postoperative complications, shorter hospital stays, a better long-term prognosis, and lower direct and indirect healthcare costs (9–11).

Although there is strong evidence to support prehabilitation programs, the definition of prehabilitation has not been standardized. As of now, it can be defined as any preoperative therapies aimed at improving patients’ physical, nutritional, medical, and mental health in order to improve their ability to tolerate surgical trauma and recover to previous conditions (12). Previously, prehabilitation programs focused solely on unimodal exercise interventions. Subsequently, recognizing that nutritional status is closely linked to inflammatory response and immune function, interventions expanded to include dietary counseling and protein supplementation (13). More recently, increasing evidence supports multimodal prehabilitation interventions, which include respiratory, aerobic, and/or resistance training programs, as well as nutritional and psychological interventions (14, 15). According to a systematic review, individuals after major abdominal surgery who received prehabilitation that involved physical exercise intervention may have lower overall morbidity and fewer postoperative pulmonary problems compared to usual care (16). Duro-Ocana et al. (3) found that supervised exercise preconditioning significantly raised peak volume of oxygen uptake (VO_2peak_), as well as 6-min walk distance (6MWD). However, the relative efficacy of different prehabilitation programs remains controversial, and evidence directly comparing these interventions is lacking, which cannot provide therapeutic effect ranking list (15). Therefore, it is important to promote the shift of prehabilitation from “experience-driven” to evidence-based individualized practice.

Network meta-analysis (NMA), which combines direct and indirect evidence, can compare the results of several interventions in the same analysis (17). It also shows the likelihood of each intervention’s relative effectiveness and allows numerous interventions to be assessed for a certain outcome, which can aid in clinical decision making (17). Consequently, the objective of our study was to investigate the optimal prehabilitation strategy for major abdominal surgery population using direct or indirect available evidence via a network meta-analysis, and provided a theoretical basis for implementing prehabilitation in clinical practice. These findings will inform the development of improved clinical research and therapeutic strategies (18).

## 2 Methods

This NMA was conducted in accordance with the Preferred Reporting Items for Systematic Reviews and Meta-Analyses for Network Meta-Analyses (PRISMA-NMA), and the study was registered in PROSPERO platform under registration number CRD42024545664.

### 2.1 Search strategy

The following databases were searched online between inception and June 2025: PubMed, Web of Science, Embase, Google Scholar, and Cochrane. The detailed search strategy was provided in Supplementary Table 1. We additionally manually searched the reference lists of the included studies and associated systematic reviews to ensure complete coverage. Furthermore, citations from specific study were checked for supplementary sources

### 2.2 Inclusion and exclusion criteria

The inclusion criteria were designed according to the PICOS principle. The inclusion criteria were as follows:

(1)    Participants: patients aged > 18 years who underwent major abdominal surgery with no restrictions on gender and surgical method, such as elective gastrointestinal, pancreatic, urological, hepatobiliary, endocrine, vascular, and abdominal transplantation surgery (3). Although the surgical procedures varied, all included studies involved major abdominal operations imposing comparable physiological stress and functional decline, thereby providing a coherent clinical context for evaluating prehabilitation’s impact on fitness and postoperative recovery; (2) Interventions: any structured prehabilitation program; (3) Comparator: control conditions included no intervention, waiting-list, usual care, health education, and one of the prehabilitation programs; (4) Outcomes: study must report one of the following outcomes, including peak volume of oxygen uptake (VO_2peak_), 6-min walk test (6MWT), length of hospital stay (LOS), and postoperative complications; (5) Study Design: randomized controlled trials (RCTs) published in any language.

The exclusion criteria were: (1) non-interventional study designs, including protocols, reviews, cohort studies, case-control studies, conference papers, and book chapters; (2) studies lacking sufficient statistical data [e.g., means, standard deviations (SDs), or sample sizes] required for effect size calculation in meta-analysis.

### 2.3 Study selection and data extraction

The study selection process was conducted independently by two reviewers using predefined inclusion and exclusion criteria. Any discrepancies between the reviewers were resolved through discussion, and a third reviewer was consulted if consensus could not be reached.

A standardized data extraction form was used to capture relevant data from each included study, including the author, year of publication, patient demographics, type of surgery, details of the prehabilitation, control conditions, and outcomes. Data from each study were extracted and converted into a format suitable for meta-analysis. When SDs were unavailable, we calculated them from standard errors (SEs), confidence intervals (CIs), t or *p*-values. For unreported data, we made at least three email attempts to contact corresponding authors. Graphical data were extracted using GetData Graph Digitizer (v2.20) when numerical results were only presented in figure format (19).

### 2.4 Interventions coding

The interventions were coded as “Prehabilitation program” or “Control.” Prehabilitation program types were identified by using the group names selected by the authors and the definitions in Table 1. It is important to acknowledge the conceptual breadth of our operational definition of “aerobic exercise.” By design, this category encompassed a range of intensities from low (e.g., walking) to moderate-vigorous (e.g., moderate-intensity continuous training, MICT). Consequently, the pooled results represent an average effect across this intensity spectrum and may mask divergent outcomes specific to either low or higher intensity training. This was a necessary compromise to facilitate a broader analysis, but it highlighted a need for more intensity-specific investigations in the future. Multimodal intervention was operationally defined as a preoperative management strategy that combined two or more distinct therapeutic modalities (e.g., exercise intervention, nutrition intervention, and/or psychological coping strategies). It is critical to note that this classification is conceptual, based on the fundamental principle of multimodal care: the potential for cumulative or synergistic effects between different components on clinical outcomes. Therefore, for the purpose of this analysis, studies meeting this combinatorial criterion were analyzed as a distinct group, irrespective of the specific parameters (such as exercise intensity) within individual components.

**TABLE 1:** Interpretation of prehabilitation program and control.

Type	Interpretation
**Control**	
Passive control	Waiting-list, no exercise/intervention provided, and keeping their lifestyle.
Active control	Standard usual care, and health education.
ERAS protocol	Standard enhanced recovery after surgery (ERAS) peri-operative care.
Physical recommendation	Advice on healthy living, including being encouraged to exercise.
**Prehabilitation program**	
High-intensity interval training (HIIT)	Characterized by repeated cycles of vigorous exercise, high-intensity interval training requires pushing the heart rate to roughly 80% of its maximum during the aerobic phase, alternating with intervals of active recovery.
Multimodal intervention	Including exercise intervention, nutrition intervention, and/or coping strategies to reduce anxiety.
Mixed exercise	At least two kinds of exercises (e.g., aerobic exercises plus resistance, HIIT plus resistance).
Aerobic exercise	Exercises that are intended to increase the capacity and efficiency of the cardiorespiratory system, such as jogging, cycling, and walking etc.

### 2.5 Risk of bias and certainty of evidence

Two independent authors assessed the methodological quality of the included RCTs using the Physiotherapy Evidence Database (PEDro) scale (20). Any discrepancies in scoring were resolved through discussion until consensus was reached. The PEDro scale consists of 11 items: eligibility criteria, random allocation, concealed allocation, baseline comparability, blinding of subjects, blinding of therapists, blinding of assessors, adequate follow-up, intention-to-treat analysis, between-group comparisons, as well as point estimates and variability. Total scores range from 0 to 10, with higher scores indicating better methodological quality. Based on the total score, studies were categorized into four quality levels: poor (< 4), fair (4–5), good (6–8), and excellent (9–10).

The overall certainty of evidence was evaluated using the Grading of Recommendations, Assessment, Development, and Evaluation (GRADE) approach (21). The certainty was potentially downgraded based on the following domains: risk of bias (study limitations), inconsistency (heterogeneity), indirectness of evidence, imprecision, and publication bias (22). Depending on the assessment across these domains, the certainty of evidence for the included studies was classified as high, moderate, low, or very low.

### 2.6 Statistical analysis

First, a pairwise meta-analysis was conducted for all outcomes to evaluate the effects of different prehabilitation interventions relative to the control group. Heterogeneity across studies was assessed using the I^2^ statistic, with I^2^ values of 25, 50, and 75% representing mild, moderate, and high heterogeneity, respectively.

Subsequently, a frequentist random-effects NMA was performed within a multivariate framework using STATA 17.0 (StataCorp, College Station, Texas, United States). This approach accounts for heterogeneity arising from clinical and methodological variations across studies and provides more conservative confidence intervals for pooled effect estimates. NMA synthesizes direct evidence (from head-to-head trials) and indirect evidence (obtained by connecting interventions via common comparators) to estimate relative effects between all interventions in a connected network, even those never directly compared. This allows for a coherent ranking of all available interventions for a given outcome. The structure of the evidence is visualized in network plots, where each intervention is represented by a node, and a line connecting two nodes indicates the presence of at least one direct comparison study.

Effect sizes were expressed as mean differences (MD) or odds ratios (OR), along with their 95% confidence intervals (95% CI), based on postintervention scores. The magnitude of the effect size was interpreted using Cohen’s criteria: values of d ≥ —0.8— were considered large, d ≥ —0.5— to < —0.8— medium, d ≥ —0.2— to < —0.5— small, and d < —0.2— trivial (23).

To ensure the transitivity of the network, clinical and methodological characteristics across studies were compared. The consistency of the network was evaluated using both global and local approaches: the design-by-treatment interaction model was applied for global inconsistency assessment, and node-splitting tests were used locally to examine discrepancies between direct and indirect evidence for each treatment contrast. The relative ranking of prehabilitation interventions was assessed using mean ranks and the surface under the cumulative ranking (SUCRA) curve. A SUCRA value of 100% indicates that an intervention is with the highest probability of being the most effective, whereas 0% suggests it is with the highest probability of being the least effective (24). Higher SUCRA values correspond to more favorable rankings.

To evaluate the robustness of the findings, sensitivity analyses were conducted by excluding studies rated as poor or fair quality. Publication bias and small-study effects were examined using funnel plots for asymmetry.

## 3 Results

### 3.1 Description of included studies

**FIGURE 1:**
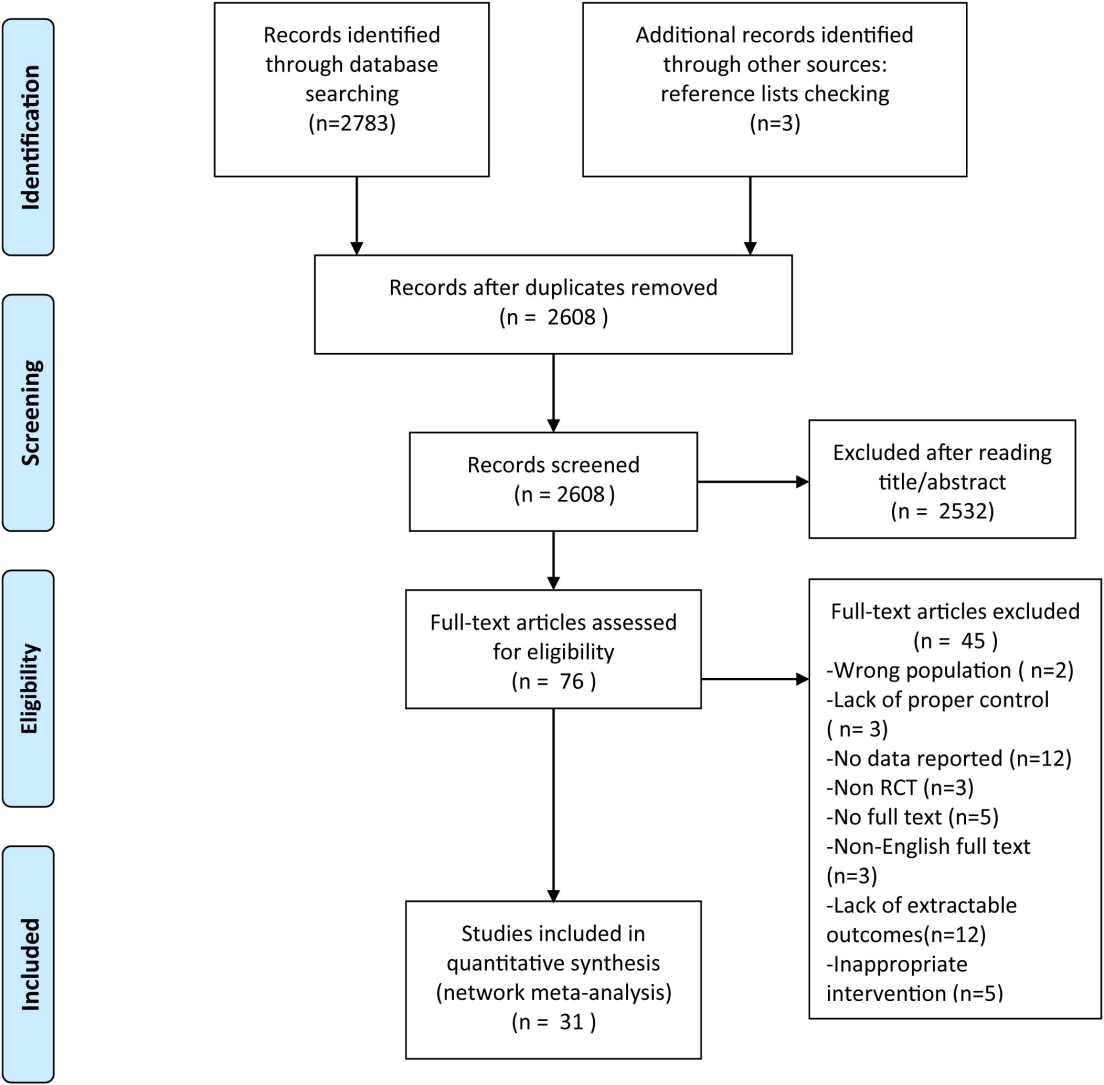
PRISMA flowchart of the study search process.

Figure 1 illustrated the study selection process according to the PRISMA flow diagram. After duplicate removal, 2,608 records were identified. Initial screening of titles and abstracts resulted in 76 potentially eligible articles. Following full-text assessment based on the inclusion and exclusion criteria, 31 studies comprising 2,467 participants were ultimately included in the network meta-analysis. The references and key characteristics of the included studies were summarized in Table 2. The studies investigated a variety of prehabilitation interventions in patients scheduled for major abdominal surgery, including colorectal, hepatobiliary, gastric, urological, and esophagogastric procedures. The prehabilitation programs were heterogeneous in design and duration, ranging from 2 to 15 weeks, with the majority implementing interventions lasting 4–6 weeks. Control groups varied widely, including passive controls (e.g., waiting-list), active controls (e.g., health education, physical activity advice), and in some cases, enhanced recovery after surgery (ERAS) protocols alone.

**TABLE 2:** Study characteristics of included studies.

First author	Prehabilitation group characteristics	Control group characteristics	Surgery	Neoadjuvant therapy	Length	Type of prehabilitation program	Type of control group
Allen et al. (25)	*N* = 26; Age: 64 ± 8 years; BMI: 28 ± 5 kg/m^2^.	*N* = 28; Age: 65 ± 6 years; BMI: 28 ± 5 kg/m^2^.	Esophagogastric cancer is planned for neoadjuvant therapy plus esophagogastrectom, or total gastrectomy.	Yes.	15 Weeks	Multimodal prehabilitation program.	Aerobic exercise such as jogging/walking/cycling.
Banerjee et al. (26)	*N* = 30; Age: 71.6 ± 6.8 years; BMI: 27.1 ± 4.2 kg/m^2^.	*N* = 30; Age:72.5 ± 8.4 years; BMI: 26.9 ± 4.5 kg/m^2^.	Bladder cancer surgery.	Yes.	3–6 Weeks	HIIT.	Advice on healthy living, including diet and being encouraged to exercise.
Barakat et al. (32)	*N* = 62; Age: 73.8 ± 6.5 years; BMI: 26.7 ± 3.5 kg/m^2^.	*N* = 62; Age: 72.9 ± 7.9 years; BMI: 27.4 ± 4.2 kg/m^2^.	Open or endovascular AAA repair.	No.	6 Weeks.	Mixed exercise.	Continue with a normal lifestyle.
Barberan-Garcia et al. (5)	*N* = 62; Age: 71 ± 11 years; BMI: 21 ± 7 kg/m^2^.	*N* = 63; Age: 71 ± 10 years; BMI: 22 ± 7 kg/m^2^.	Elective major abdominal surgery	No.	6 Weeks.	HIIT.	Advice on healthy living, including diet and being encouraged to exercise.
Berkel et al. (58)	*N* = 28; Age: 74 ± 7 years; BMI: 29.8 ± 4.1 kg/m^2^.	*N* = 29; Age: 73 ± 6 years.; BMI: 30.5 ± 4.9 kg/m^2^.	Colorectal cancer.	Yes.	3 Weeks.	Mixed exercise.	Nutritional counseling and advice on smoking cessation.
Bousquet-Dion et al. (34)	*N* = 41; Age: 74(67.5–78) years BMI: 27.5 ± 4.1 kg/m^2^.	*N* = 39; Age: 71(54.5–74.5) years; BMI: 28.6 ± 4.5 kg/m^2^.	Colon or rectal cancer resection.	No.	4 Weeks.	Multimodal prehabilitation program.	ERAS protocol.
Dronkers et al. (59)	*N* = 22; Age: 71.1 ± 6.3 years; BMI: 26.6 ± 3.6 kg/m^2^.	*N* = 20; Age: 68.8 ± 6.4 years; BMI 25.7 ± 3.1 kg/m^2^.	Elective colon surgery.	No.	2–4 Weeks.	Mixed exercise.	Active for minimally 30 min a day
Dunne et al. (27)	*N* = 20; Age: 61 (56–66) years; BMI: 29.7 ± 4.2 kg/m^2^.	*N* = 18; Age: 62(53–72) years; BMI: 29.3 ± 4.2 kg/m^2^.	Colorectal liver metastasis resection.	Yes.	4 Weeks.	HIIT.	Follow clinical advice on home exercise
Fulop et al. (36)	*N* = 77; Age: 70 (60–75) years; BMI: 27.9 ± 5.6 kg/m^2^.	*N* = 72; Age: 70 (64–75) ears; BMI: 27.9 ± 5.3 kg/m^2^.	Colorectal surgery.	No.	3–6 Weeks.	Multimodal prehabilitation program.	ERAS protocol.
Blackwell et al. (28)	*N* = 19; Age: 71 ± 2 years.; BMI: not reported.	*N* = 21; Age: 72 ± 4 years; BMI: not reported.	Urological cancer.	No.	4 Weeks.	HIIT.	Maintain their habitual physical activity and dietary
Gloor et al. (60)	*N* = 54; Age: 66 (24–90) years.; BMI: 26(20–35) kg/m^2^.	*N* = 53; Age: 65 (29–86) years; BMI: 27 (18–40) kg/m^2^.	Colorectal resection.	No.	3–6 Weeks.	HIIT, resistance	Patients were encouraged to remain physically active
Kaibori et al. (4)	*N* = 25; Age: 68.0 ± 9.1 years.; BMI: not reported.	*N* = 26; Age: 71.3 ± 8.8 years; BMI: not reported.	Liver resection.	No.	1 Month.	Walking, stretching exercises.	Management with diet.
Karlsson et al. (61)	*N* = 10; Age: 83.5 (76–85) years.; BMI: not reported.	*N* = 10; Age: 74.0 (73–76) years.; BMI: not reported.	Colorectal cancer surgery.	Yes.	2 Weeks.	Mixed exercise.	Recommendation of 150 min/week of moderate physical activity.
Kim et al. (33)	*N* = 14; Age: 55 ± 15 years.; BMI: 26.6 ± 5.9 kg/m^2^.	*N* = 7; Age: 65 ± 9 years.; BMI: 25.3 ± 2.7 kg/m^2^.	Colo-rectal surgery.	No.	4 Weeks.	Aerobic exercise training.	Basic instructions to prepare for surgery, without an exercise prescription.
Northgraves et al. (40)	*N* = 11; Age: 64.1 ± 10.5 years; BMI: 30.3 ± 4.3 kg/m^2^.	*N* = 11; Age: 63.5 ± 12.5 years.; BMI: 27.8 ± 5.7 kg/m^2^.	Elective cancer colorectal surgery.	Yes.	22 ± 7.5 Days.	Mixed exercise.	Maintain normal exercise levels.
Soares et al. (42)	*N* = 18; Age: 58.5 (51.3–63.5) years; BMI: 23.6 (19.7–25.9) kg/m^2^.	*N* = 19; Age: 55.0 (49.3–64.3) years; BMI: 24.2 (21.3–28.4) kg/m^2^.	Elective open abdominal Surgery.	No.	2–3 Weeks.	Walking, inspiratory muscle training.	Not receive any physical therapy.
Steffens et al. (62)	*N* = 11; Age: 62 (48–72) years; BMI: not reported.	*N* = 11; Age: 66 (46–70) years; BMI: not reported.	Pelvic exenteration.	No.	2–6 Weeks.	Mixed exercise.	Nutritional counseling and advice on smoking cessation and reduction of alcohol intake.
Tew et al. (29)	*N* = 27; Age: 74.6 ± 5.5 years; BMI: 26.5 ± 4.1 kg/m^2^.	*N* = 26; Age 74.9 ± 6.4 years; BMI: 26.8 ± 3.4 kg/m^2^.	Open or endovascular repair of an infrarenal AAA.	No.	4 Weeks.	HIIT.	Usual care.
Waller et al. (37)	*N* = 11; Age: 55.5 (49.2, 61.7) years; BMI: 30.0 (25.6,34.4) kg/m^2^.	*N* = 11; Age: 61.0 (53.1, 68.9) years; BMI: 27.8(23.4, 32.2) kg/m^2^.	Major abdominal cancer surgery at a tertiary cancer.	No.	≥ 2 Weeks.	Multimodal prehabilitation program.	No prehabilitation program.
West et al. (47)	*N* = 22; Age: 64 (45 –82) years; BMI: not reported.	*N* = 13; Age: 64 (45 –82) years; BMI: not reported.	Rectal cancer surgery after NACRT.	Yes.	6 Weeks.	HIIT.	No exercise intervention.
Woodfield et al. (31)	*N* = 28; Age: 66.5 (13.5) years; BMI: Not reported.	*N* = 35; Age: 66 (15) years; BMI: Not reported.	Major abdominal surgery.	No.	4 Weeks.	HIIT.	Encourage them to exercise more before surgery.
Carli et al. (35)	*N* = 55; Age: 78 (72–82) years.; BMI: 24.9 (23–30.1) kg/m^2^.	*N* = 55; Age: 82 (75–84) years; BMI: 26.4 (23.8–30.6) kg/m^2^.	Non-metastatic colorectal cancer.	No.	4 Weeks.	Multimodal prehabilitation program.	Advice on smoking and alcohol cessation.
Carli et al. (41)	*N* = 58; Age: 61 (16) years; BMI: 28(6) kg/m^2^.	*N* = 54; Age: 60 (15) years; BMI: 27 (5) (*n* = 53) kg/m^2^.	Colorectal surgery.	Yes.	3–6 Weeks.	Mixed exercise.	Walk.
Gills et al. (38)	*N* = 38; Age: 65.7 (13.6) years; BMI: 26.9 (4.6) kg/m^2^.	*N* = 39; Age: 66.0 (9.1) years; BMI: 28.5 (4.3) kg/m^2^.	Colorectal resection.	Yes.	4 Weeks.	Multimodal prehabilitation program.	Not receive any intervention before surgery.
Minnella et al. (39)	*N* = 26; Age: 67.3 (7.4) years; BMI: 26.1 (4.8) kg/m^2^.	*N* = 25; Age: 68.0 (11.6) years; BMI: 25.7 (4.7) kg/m^2^.	Elective esophagogastric resection.	Yes.	Not reported.	Multimodal prehabilitation program.	EARS.
Jensen et al. (63)	*N* = 50; Age: 69 (66–72) years; BMI: 26 (25–27) kg/m^2^.	*N* = 57; Age: 71 (68–73) years; BMI: 26 (25–27) kg/m^2^.	Radical cystectomy.	No.	2 Weeks.	Mixed exercise.	Lifestyle, nutritional status, and physical activity.
Minnella et al. (39)	*N* = 35; Age: 69.7 (10.2) years; BMI: 26.4 (4.0) kg/m^2^.	*N* = 35; Age: 66.0 (10.2) years; BMI: 28.4 (5.4) kg/m^2^.	Elective radical cystectomy.	Yes.	Not reported.	Multimodal prehabilitation program.	Standard perioperative care.
Onerup et al. (64)	*N* = 317; Age: 69 ± 11 years; BMI: 26 ± 4.2 kg/m^2^.	*N* = 351; Age: 68 ± 11 years; BMI: 26 ± 4.8 kg/m^2^.	Colorectal cancer surgery.	Yes.	14 ± 4 Days	Aerobic activity, Inspiratory muscle training	Usual care.
Bausys et al. (12)	*N* = 61; Age: 61 (11) years; BMI: 25.5 (5.4) kg/m^2^.	*N* = 61; Age: 64 (10) years; BMI: 27.1 (4.9) kg/m^2^.	Elective gastric cancer surgery or surgery after neoadjuvant chemotherapy.	Yes.	4 Weeks.	Multimodal prehabilitation program.	Patients were recommended to use high-energy nutritional supplements.
Moug et al. (43)	*N* = 24; Age: 65.2 (11.4) years; BMI: 26.5 (3.0) kg/m^2^.	*N* = 24; Age: 66.5 (9.6) years; BMI: 28.0 (3.4) kg/m^2^.	Potentially curative surgery.	Yes.	13 Weeks.	Walk.	Standard care.
Daniesson et al. (65)	*N* = 27; Age: 78(4) years; BMI: None.	*N* = 24; Age: 77(7) years; BMI: None.	Resection of colorectal adenocarcinoma and/or colorectal liver metastases	Yes.	2–3 Weeks.	Multimodal prehabilitation program.	Recommendation to perform moderate intensity.

### 3.2 Methodological quality

The overall methodological quality of the included studies, as assessed by the PEDro scale, was consistently good, with scores ranging from 6 to 10 (Supplementary Table 2). Due to the inherent challenges associated with blinding in exercise-based interventions, full blinding of participants and personnel was limited across studies. Specifically, therapists were blinded in three studies, participants were blinded in three studies, and outcome assessors were blinded in the majority of the studies.

### 3.3 Primary outcomes

**FIGURE 2:**
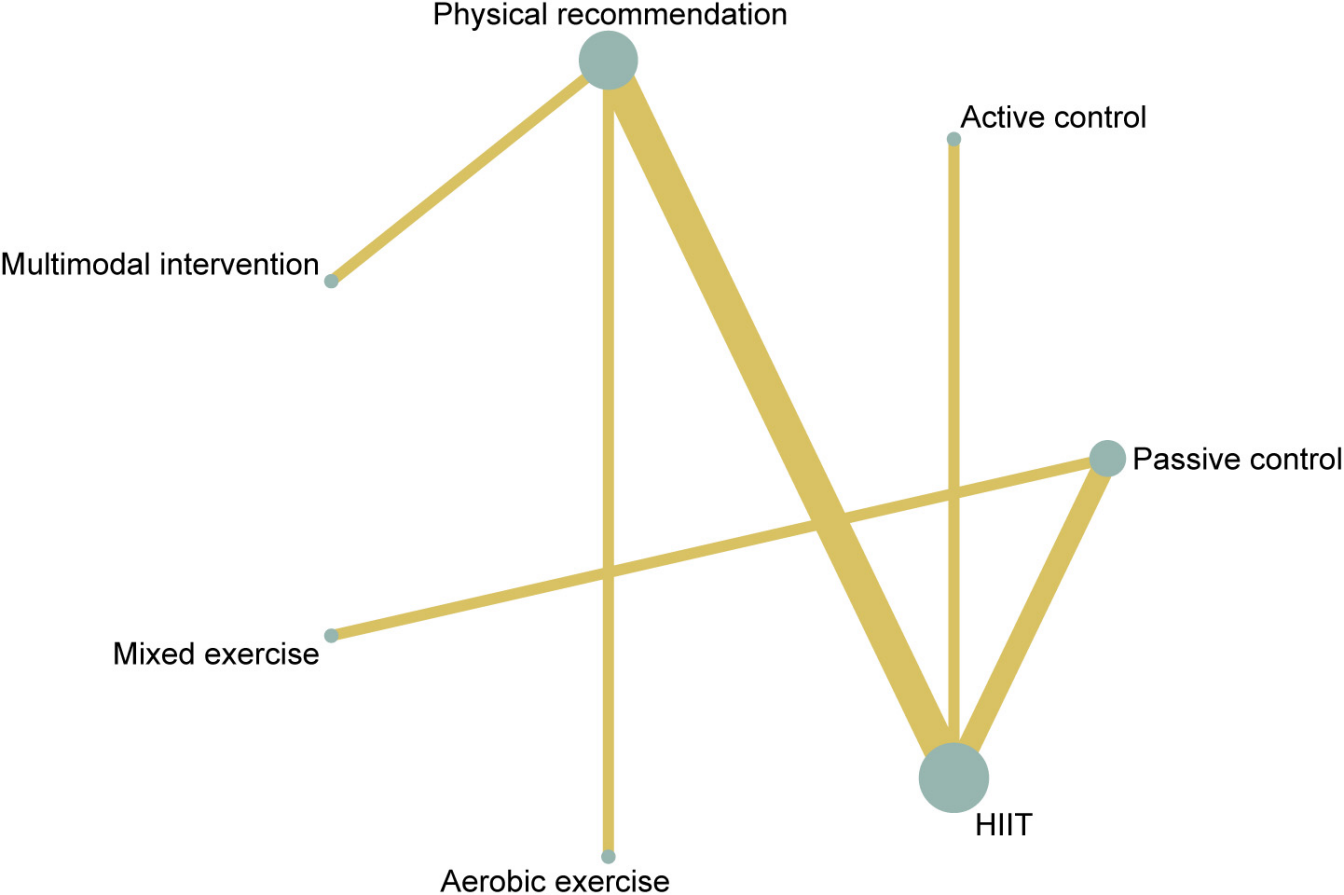
Network plot of comparisons and the efficacy of varied treatments compared with the control group for VO_2peak_. VO_2peak_, peak volume of oxygen uptake.

**FIGURE 3:**
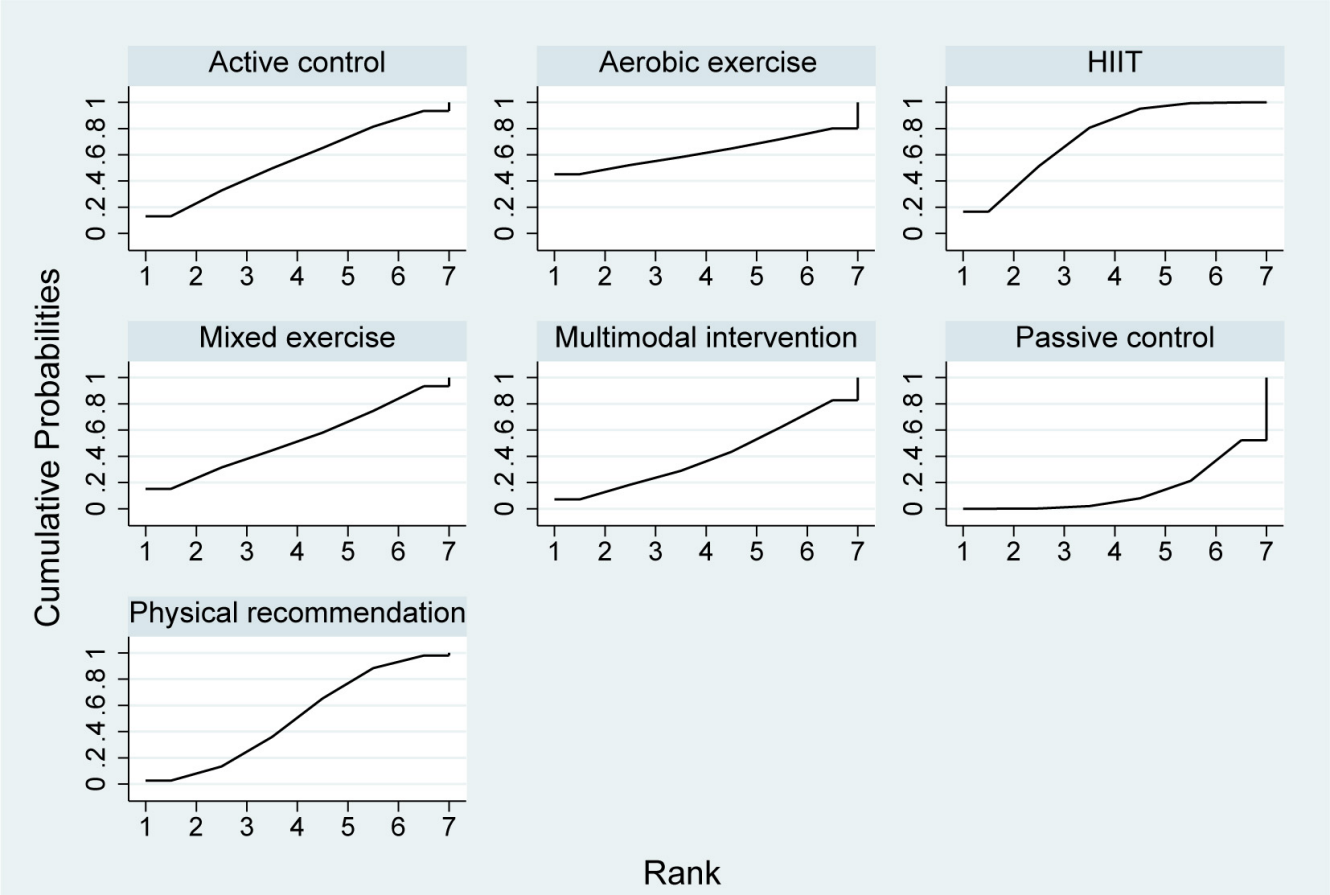
Cumulative ranking probability plots for VO_2peak_. VO_2peak_, peak volume of oxygen uptake.

A total of ten studies involving 361 participants were included in the analysis of VO_2peak_. Among these, one study evaluated a multimodal prehabilitation program (25), six investigated HIIT (26–31), one assessed mixed exercise (32) and two examined aerobic exercise (25, 33) (Table 2). The paired meta-analysis demonstrated that prehabilitation significantly improved VO_2peak_ compared with the control group (MD = 1.66, 95% CI: 1.01–2.32, I^2^ = 11.4%) (Supplementary Figure 1). The network plot of VO_2peak_ (Figure 2) illustrated all available treatment comparisons. Network meta-analysis indicated that only HIIT was significantly superior to passive controls (SUCRA = 73.9%, MD = 2.29, 95% CI: 0.52–4.06). The relative effectiveness of all interventions was detailed in Table 3. Ranking based on SUCRA values and cumulative probability curves was provided in Supplementary Table 3 and Figure 3. Comparison-adjusted funnel plots showed no obvious evidence of publication bias. The certainty of evidence for these outcomes was rated from moderate to very low (Supplementary Table 4).

**TABLE 3:** Comparative effectiveness results for VO_2peak_.

HIIT						
−0.24 (−6.90, 6.43)	Aerobic exercise					
0.50 (−1.82, 2.82)	0.74 (−6.32,7.80)	Active control				
0.69 (−2.30, 3.68)	0.93 (−6.38, 8.23)	0.19 (−3.59, 3.98)	Mixed exercise			
0.76 (−0.95, 2.48)	1.00 (−5.44,7.44)	0.26 (−2.63, 3.16)	0.07 (−3.37, 3.52)	Physical recommendation		
1.20 (−1.83,4.24)	1.44 (−5.47,8.35)	0.70 (−3.12,4.53)	0.51 (−3.75,4.77)	0.44 (−2.06, 2.94)	Multimodal intervention	
2.29 (0.52, 4.06)	2.53 (−4.37, 9.42)	1.79 (−1.13, 4.71)	1.60 (−0.81, 4.01)	1.53 (−0.93, 3.99)	1.09 (−2.42, 4.60)	Passive control

Each cell shows an MD with a 95%CI. VO_2peak_, maximal oxygen consumption; 95%CI = 95% confidence interval. The entire table presents the statistical results of pairwise comparisons between interventions. Cells highlighted in dark color indicate statistically significant differences.

**FIGURE 4:**
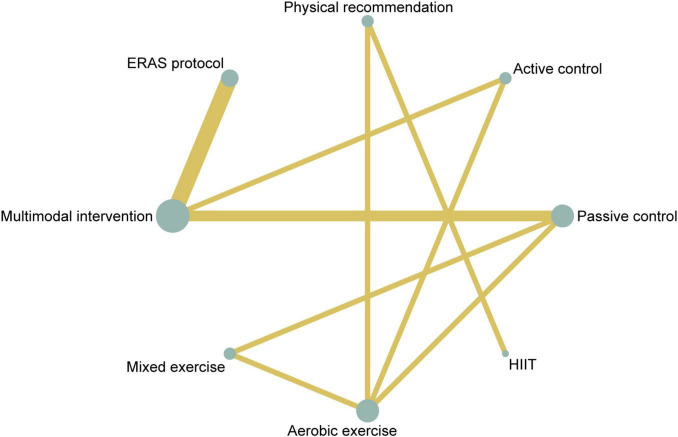
Network plot of comparisons and the efficacy of varied treatments compared with the control group for 6MWT. 6MWT, 6-min walk test.

**FIGURE 5:**
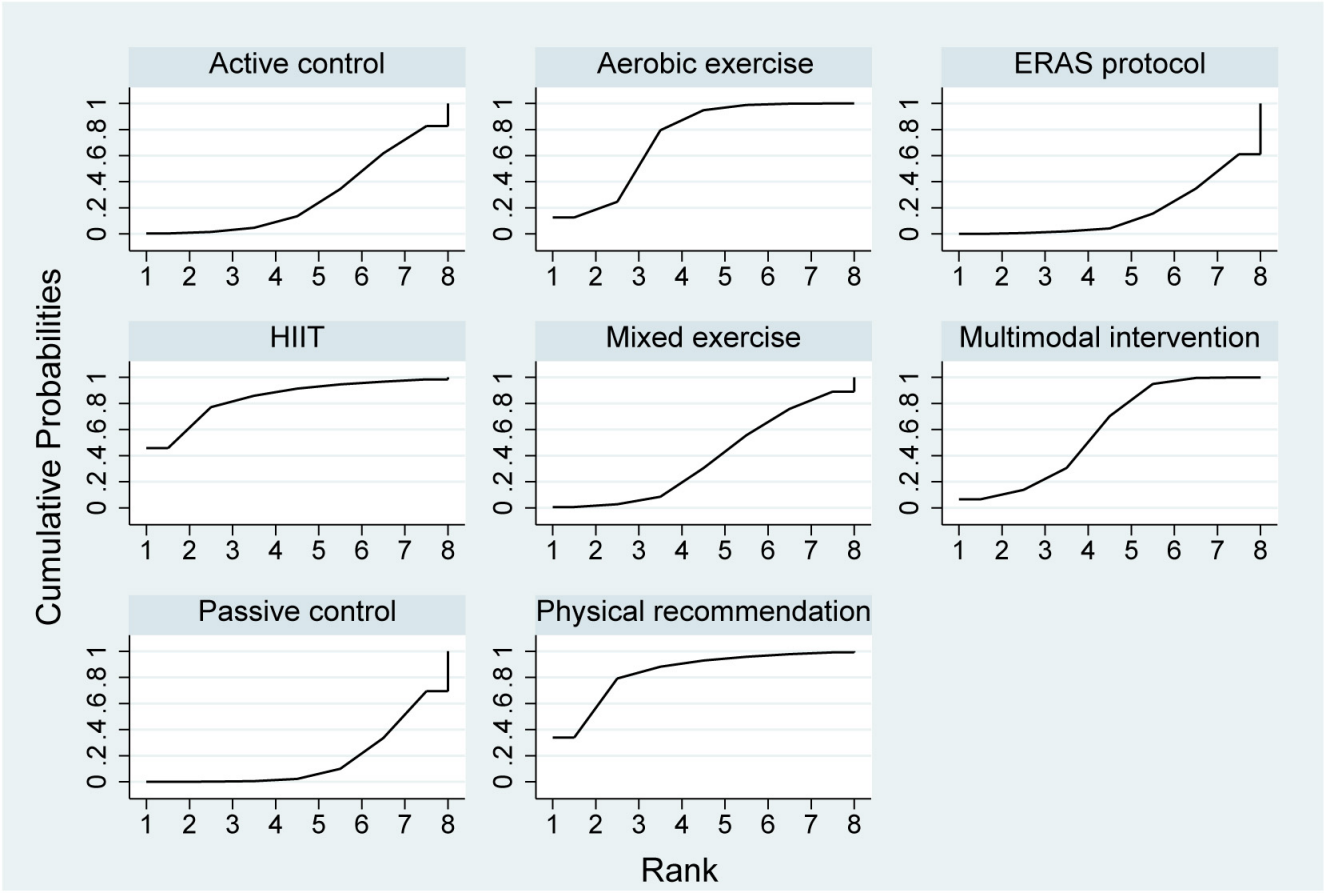
Cumulative ranking probability plots for 6MWT. 6MWT, 6-min walk test.

A total of 13 studies involving 856 participants were included in the analysis of 6MWT. Among these, six studies evaluated multimodal prehabilitation programs (34–39), one investigated HIIT (5), two assessed mixed exercise (40, 41) and four examined aerobic exercise (33, 41–43; Table 2). Pairwise meta-analysis indicated that prehabilitation interventions significantly improved 6MWT performance compared to control groups, with an overall *I*^2^ value of 36.8% (Supplementary Figure 2). The network plot (Figure 4) confirmed that all prehabilitation modalities were directly compared to passive controls. Network meta-analysis revealed that both aerobic exercise (SUCRA = 72.9%, MD = 71.67, 95% CI: 17.44–125.90) and multimodal intervention (SUCRA = 59.4%, MD = 48.51, 95% CI: 3.52–93.50) were significantly more effective than control conditions (Table 4). Ranking of interventions based on SUCRA values and cumulative probabilities were illustrated in Figure 5 and detailed in Supplementary Table 3. Comparison-adjusted funnel plots showed no substantial evidence of publication bias (Supplementary Figure 3). The overall certainty of evidence for 6MWT outcomes was rated as moderate to very low (Supplementary Table 5).

**TABLE 4:** Comparative effectiveness results for 6MWT.

HIIT							
4.00 (−47.16, 55.16)	Physical recommendation						
41.00 (−67.18, 149.18)	37.00 (−58.31, 132.31)	Aerobic exercise					
64.16 (−61.57, 189.89)	60.16 (−54.69, 175.01)	23.16 (−40.92, 87.24)	Multimodal intervention				
85.88 (−34.30, 206.07)	81.88 (−26.86, 190.63)	44.88 (−7.48, 97.25)	21.72 (−52.22, 95.67)	Mixed exercise			
99.35 (−29.18, 227.88)	95.35 (−22.56, 213.26)	58.35 (−11.06, 127.77)	35.19 (−18.82, 89.21)	13.47 (−67.15, 94.09)	Active control		
113.90 (−17.26, 245.06)	109.90 (−10.87, 230.67)	72.90 (−1.27, 147.07)	49.74 (14.66, 84.82)	28.02 (−55.24, 111.28)	14.55 (−50.61, 79.71)	ERAS protocol	
112.67 (−8.34, 233.68)	108.67 (−0.99, 218.33)	71.67 (17.44, 125.90)	48.51 (3.52, 93.50)	26.79 (−35.98, 89.55)	13.32 (−49.99, 76.62)	−1.23 (−60.13, 57.67)	Passive control

Each cell shows an MD with a 95%CI. 6MWT, 6-min walk test; 95%CI = 95% confidence interval. The entire table presents the statistical results of pairwise comparisons between interventions. Cells highlighted in dark color indicate statistically significant differences.

### 3.4 Secondary outcomes

Twenty-three trials comprising 2204 patients assessing LOS were included in the network meta-analysis. Pairwise meta-analysis indicated that prehabilitation interventions were associated with a reduction in LOS, with heterogeneity of *I*^2^ = 2.6% (Supplementary Figure 4A). The network plot was illustrated in Supplementary Figure 5A. Network meta-analysis indicated that only multimodal intervention significantly reduced LOS compared to control (SUCRA = 74.7%, MD = –1.50, 95% CI: –3.02 to –0.02) (Supplementary Table 7). Ranking of interventions based on SUCRA values and cumulative probabilities were presented in Supplementary Figure 6A and Supplementary Table 6. Comparison-adjusted funnel plots did not reveal clear evidence of publication bias (Supplementary Figure 7A). The overall certainty of the evidence was rated as moderate to very low (Supplementary Table 9).

Fifteen trials involving 861 patients reported on postoperative complications. Pairwise meta-analysis showed a beneficial overall effect of prehabilitation on reducing postoperative complications, with moderate heterogeneity (*I*^2^ = 56.3%; Supplementary Figure 4B). The network plot was shown in Supplementary Figure 5B. HIIT showed a significant reduction versus passive control (SUCRA = 98.4%, OR = 0.03, 95% CI: 0.00–0.51) (Supplementary Tables 6, 8). Ranking of interventions based on cumulative probability plots was provided in Supplementary Figure 6B. Corrected funnel plots did not indicate substantial publication bias (Supplementary Figure 7B). The certainty of evidence ranged from high to very low across comparisons (Supplementary Table 10).

## 4 Discussion

We conducted a network meta-analysis to compare the relative effectiveness of various prehabilitation interventions on VO_2peak_, 6MWT, LOS, and postoperative complications in patients undergoing major abdominal surgery. The analysis incorporated 31 studies involving 2,467 participants. To our knowledge, this is the first and most comprehensive network meta-analysis evaluating the impact of diverse prehabilitation regimens on these surgical outcomes within this patient population. The findings demonstrate that prehabilitation strategies confer benefits in improving VO_2peak_ and 6MWT. HIIT emerged as the most effective intervention for enhancing preoperative VO_2peak_ and reducing postoperative complications. Aerobic exercise was associated with the greatest improvement in 6-min walk distance, with multimodal interventions also showing significant efficacy in this outcome. Notably, multimodal prehabilitation was further found to significantly reduce LOS. Given the limited quality and variability of direct evidence among the included studies, these results should be interpreted with caution.

HIIT is characterized by alternating short bursts of high-intensity exercise with periods of recovery or low-intensity activity. Studies conducted in both athletic and general populations have demonstrated that elevating exercise intensity enhances the training stimulus and promotes adaptations such as improved performance, anaerobic threshold (AT), and maximal oxygen consumption (VO_2m*ax*_) (44). Due to current treatment standards, the time between a cancer diagnosis and surgery is typically limited (e.g., 34 days maximum for colorectal cancer) (45). Therefore, a short-term, effective preoperative physical exercise training program is required (45). In the studies included in our analysis, HIIT programs were implemented over periods of ≥ 3 weeks. HIIT is increasingly recognized within prehabilitation contexts as a time-efficient training modality that rapidly enhances aerobic fitness, offering a physiologically viable strategy for optimizing preoperative cardiopulmonary capacity in a limited timeframe.

Maximal oxygen consumption, assessed via cardiopulmonary exercise testing (CPET), serves as an objective measure of cardiopulmonary health and a prognostic indicator for postoperative morbidity, cardiovascular events, and all-cause mortality following abdominal surgery (46). Improvements in VO_2m*ax*_ reflect positive adaptations to training; a meta-analysis indicated that an increase of 3.5 ml/min/kg in VO_2m*ax*_ is associated with a 13–15% reduction in cardiovascular and all-cause mortality (46). For a surgical population, this level of improvement could plausibly translate into a lower risk of postoperative complications and enhanced recovery, although future prospective studies are needed to confirm this causal link directly. The study determined that the best CPET cut-offs to identify patients who may have higher postoperative morbidity were < 18.6 ml/min/kg of VO_2peak_ and 10.6 ml/min/kg of VO_2_AT (47). Given that surgery often cannot be delayed, effective prehabilitation must achieve clinically meaningful gains within a short duration. Mechanistic studies suggest that HIIT improves VO_2peak_ through upregulation of PGC-1α, which enhances mitochondrial biogenesis and aerobic capacity (48). Additionally, HIIT has been shown to increase the rate of Ca^2+^ reuptake into the sarcoplasmic reticulum by 50–60%, improving calcium cycling efficiency, reducing skeletal muscle fatigue, and ultimately supporting gains in cardiorespiratory fitness (CRF) (48, 49).

Preoperative training has been shown to enhance functional capacity, with previous research indicating that the 6MWD is a strong predictor of postoperative morbidity, and that VO_2peak_ is closely correlated with functional performance as measured by the 6MWT (50). The 6MWT assesses the maximum distance a patient can walk in 6 min and is considered both easy to administer and more reflective of activities of daily living compared to other walking tests (51). In the present study, patients who underwent prehabilitation involving aerobic or walking-based exercises showed greater improvement in 6MWD compared to those receiving usual care. Enhancing preoperative walking capacity through targeted training may contribute to reduced postoperative morbidity. Although HIIT significantly improved VO_2peak_, it did not yield a statistically significant increase in 6MWD. This discrepancy may be attributed to the fact that none of the HIIT interventions incorporated walking-specific activities. In contrast, Bhatia et al. (52) reported a median increase of 20% (14–26%) in 6MWD among lung cancer patients following a combined intervention that included both HIIT and encouraged walking with pedometer use. Most notably, multimodal prehabilitation was associated with substantial improvements in preoperative 6MWT performance. This aligns with previous reports on multimodal rehabilitation protocols (14, 53), and suggests that the integration of exercise with nutritional support enhances muscle strength and functional reserve, thereby explaining the superior outcomes (54).

Our meta-analysis supports the beneficial effects of prehabilitation exercise on reducing LOS and postoperative complications. A previous meta-analysis suggested that prehabilitation may improve surgical outcomes in high-risk cancer patients (55), demonstrating a significant reduction in major complications (RR = 0.09, 95% CI: –0.15 to –0.03, *p* = 0.005) and LOS (MD = –2.7, 95% CI: –5.37 to –0.17, *p* = 0.04) compared with standard care (55). Due to variability in complication classifications across studies, we analyzed the overall incidence of all complication types. While some earlier meta-analyses reported significantly fewer complications in prehabilitation groups compared to usual care (6, 16), one larger study incorporating both supervised and unsupervised interventions found no significant improvement (14). Unlike previous work, the current analysis did not differentiate between exercise modalities in relation to their effects on complications, which may also reflect differences in the number and design of included studies. LOS is widely used as a postoperative endpoint and is considered an indicator of quality care (3). In our study, only multimodal prehabilitation was associated with a statistically significant reduction in LOS. However, the possibility of bias due to the limited number of studies for certain intervention types cannot be excluded, and the efficacy of other prehabilitation modes in shortening LOS warrants further investigation.

The findings of this meta-analysis hold important implications for both future research and clinical practice. Across various clinical contexts, a change in VO_2peak_ equivalent to 1 metabolic equivalent (MET) (3.5 mL/kg/min) is considered the minimal clinically important difference (MCID) (56). Although HIIT resulted in greater improvements in VO_2peak_ compared to usual care, the mean change did not exceed this MCID threshold. In contrast, the observed mean increase in 6MWD of 71.67 meters following prehabilitation exceeded the established MCID for 6MWD (> 20 m) (57). It is important to note that the waiting time for surgery may extend beyond 12 weeks in patients receiving neoadjuvant chemotherapy or radiotherapy for malignancies such as ovarian, colorectal, bladder, or gastric cancers (8). Prehabilitation programs should ideally be offered to patients at least 2 weeks prior to surgery, with a preferred window of 4–6 weeks to allow adequate time for intervention efficacy (8). Although the preoperative period is often limited, we recommend implementing exercise interventions before surgery to maximize functional gains. Furthermore, we advocate for enhanced health education efforts by healthcare providers, supported by home-based monitoring and guided training, to facilitate an extended and effective prehabilitation timeframe.

Several limitations of this network meta-analysis should be acknowledged. First, despite including only RCTs, residual clinical heterogeneity may stem from variations in prehabilitation protocols, supervision intensity, and the mix of surgical procedures. Second, due to insufficient data, we were unable to perform subgroup analyses to explore potential differential effects of supervised versus unsupervised programs, or to examine specific types of postoperative complications. Third, the strength of evidence for several comparisons was low or very low, urging caution in interpretation. Specific to the NMA methodology, while the network was sufficiently connected for analysis, the scarcity of direct head-to-head comparisons between different active prehabilitation types meant that many rankings relied heavily on indirect evidence, which may be less robust. Furthermore, the categorization of complex, multimodal interventions into a single node, while necessary for analysis, may obscure the specific contribution of individual components (e.g., nutrition vs. psychological support). Finally, the potential for performance bias exists as blinding of participants and personnel was often not feasible in exercise trials.

## 5 Conclusion

In conclusion, this network meta-analysis suggests that the optimal prehabilitation strategy prior to major abdominal surgery may depend on the targeted outcome, with HIIT being the most effective strategy for improving VO_2peak_ and reducing postoperative complications, aerobic exercise providing the greatest benefit for 6MWD, and multimodal interventions offering the strongest advantage for reducing LOS. However, due to the limited head-to-head evidence between interventions, we refrain from concluding that a specific program must be selected to achieve a single specific goal. Future clinical decision-making should carefully weigh the promising evidence from this study (e.g., HIIT for cardiorespiratory improvement, multimodal intervention for potential LOS reduction), individual patient characteristics, functional baseline, and available local resources to develop a comprehensive prehabilitation strategy.

## Data Availability

The raw data supporting the conclusions of this article will be made available by the authors, without undue reservation.
