# Supplementary material for: Effects of different prehabilitation programs on the major abdominal surgery population: a systematic review and network meta-analysis
Source: Front Med (Lausanne). 2026 Jan 14;12:1673338. doi: 10.3389/fmed.2025.1673338 (PMC12846943; doi:10.3389/fmed.2025.1673338)
Supplement: Supplementary file 1 [file Supplementary_file_1.docx]

| Step | Search strategy |
| --- | --- |
| #1 | Search ((“abdominal surgery[Mesh Terms])”) OR ((((((((((((((((((((((((“digestive system surgical procedure” OR “colorectal cancer”) OR “colorectal neoplasm”) OR “colorectal surgery”) OR “bowel cancer”) OR “bowel surgery”) OR “bowel neoplasm”) OR “colon cancer”) OR “colon neoplasm”) OR “colon surgery”) OR “liver cancer”) OR “liver neoplasm”) OR “liver surgery”) OR “liver transplantation”) OR “hepatic cancer”) OR “hepatic surgery”) OR “hepatic neoplasm”) OR “hepatic transplantation”) OR “pancreatic cancer”) OR “pancreatic surgery”) OR “pancreatic neoplasm”) OR “pancreas cancer”) OR “pancreas surgery”) OR “pancreas neoplasm”)) |
| #2 | Search (exercise [Mesh Terms]) OR ((((((((((exercises [Title/Abstract]) OR “physical activity” [Title/Abstract]) OR training*[Title/Abstract]) OR prehabilitation [Title/Abstract]) OR “physical intervention” [Title/Abstract]) OR “physical therapy” [Title/Abstract]) OR “Exercise intervention” [Title/Abstract]) OR “exercise program” [Title/Abstract]) OR “exercise therapy” [Title/Abstract]) |
| #3 | Search ((randomized controlled trial [pt] OR controlled clinical trial [pt] OR randomized [tiab] OR clinical trials as topic [mesh: noexp] OR randomly [tiab] OR trial [ti]) NOT (animals [mh] NOT humans [mh])) |
| #4 | Search(“morbidity”) OR “morbidities”) OR “complication”) OR “complications”) OR length of stay [Mesh Terms]) OR “hospitalization”) OR walk test [Mesh Terms]) OR “6-minutes-walk”) OR “6MWT”) OR “6MWD”) OR “VO_2 peak_”) OR “VO_2max”_) OR “oxygen consumption”) OR “CPET”) OR “cardiopulmonary test”) OR “maximal oxygen uptake”) OR “maximum oxygen uptake”) |
| #5 | #1 AND #2 AND #3 AND #4 |

**Table 1 Search strategy in PubMed**

**Table 2 Quality assessment for** **RCTs**

| Reference | eligibility criteria | random allocation | concealed allocation | baseline comparability | blind subjects | blind therapists | blind assessors | adequate follow-up | intention-to-treat analysis | between-group comparisons | point estimates and variability | Total  (0-10) |
| --- | --- | --- | --- | --- | --- | --- | --- | --- | --- | --- | --- | --- |
| Allen et al.2022 | 1 | 1 | 1 | 1 | 0 | 0 | 1 | 1 | 1 | 1 | 1 | 8 |
| Banerjee et al.2017 | 1 | 1 | 1 | 1 | 0 | 1 | 1 | 1 | 1 | 1 | 1 | 9 |
| Barakat et al.2016 | 1 | 1 | 1 | 1 | 0 | 0 | 1 | 1 | 1 | 1 | 1 | 8 |
| Barberan-Garcia et al.2017 | 1 | 1 | 1 | 1 | 0 | 0 | 1 | 1 | 1 | 1 | 1 | 8 |
| Berkel et al.2022 | 1 | 1 | 1 | 1 | 1 | 0 | 0 | 0 | 1 | 1 | 1 | 7 |
| Bousquet-Dion et al.2018 | 1 | 1 | 1 | 1 | 0 | 0 | 1 | 0 | 1 | 1 | 1 | 7 |
| Carli et al.2020 | 1 | 1 | 1 | 1 | 0 | 0 | 1 | 1 | 1 | 1 | 1 | 8 |
| Dronkers et al.2010 | 1 | 1 | 1 | 1 | 0 | 0 | 1 | 1 | 0 | 1 | 1 | 7 |
| Dunne et al.2016 | 1 | 1 | 1 | 1 | 0 | 0 | 1 | 1 | 0 | 1 | 1 | 7 |
| Fulop et al.2021 | 1 | 1 | 1 | 1 | 0 | 0 | 1 | 0 | 0 | 1 | 1 | 6 |
| Blackwell et al.2020 | 1 | 1 | 1 | 1 | 0 | 0 | 1 | 1 | 0 | 1 | 1 | 7 |
| Gloor et al.2022 | 1 | 1 | 1 | 1 | 0 | 0 | 0 | 1 | 1 | 1 | 1 | 7 |
| Kaibori et al.2013 | 1 | 1 | 0 | 1 | 0 | 0 | 0 | 1 | 1 | 1 | 1 | 6 |
| Karlsson et al.2019 | 1 | 1 | 0 | 1 | 0 | 0 | 1 | 1 | 1 | 1 | 1 | 7 |
| Kim et al.2009 | 1 | 1 | 0 | 1 | 0 | 0 | 0 | 1 | 1 | 1 | 1 | 6 |
| Northgraves et al.2019 | 1 | 1 | 1 | 1 | 0 | 0 | 0 | 1 | 1 | 1 | 1 | 7 |
| Soares et al.2013 | 1 | 1 | 1 | 1 | 0 | 0 | 0 | 1 | 1 | 1 | 1 | 7 |
| Steffens et al.2021 | 1 | 1 | 1 | 1 | 0 | 1 | 1 | 1 | 0 | 1 | 1 | 9 |
| Tew et al.2017 | 1 | 1 | 1 | 1 | 0 | 0 | 1 | 1 | 0 | 1 | 1 | 8 |
| Waller et al.2021 | 1 | 1 | 1 | 1 | 0 | 0 | 0 | 1 | 0 | 1 | 1 | 7 |
| West et al.2014 | 1 | 1 | 0 | 0 | 0 | 0 | 1 | 1 | 0 | 1 | 1 | 6 |
| Woodfield et al.  2021 | 1 | 1 | 1 | 1 | 0 | 0 | 1 | 1 | 1 | 1 | 1 | 9 |
| Carli et al.2010 | 1 | 1 | 0 | 0 | 0 | 0 | 0 | 1 | 1 | 1 | 1 | 6 |
| Gills et al.2014 | 1 | 1 | 1 | 1 | 0 | 0 | 1 | 1 | 1 | 1 | 1 | 9 |
| Minnella et al  2018 | 1 | 1 | 1 | 1 | 0 | 0 | 1 | 1 | 1 | 1 | 1 | 9 |
| Jensen et al.2014 | 1 | 1 | 0 | 1 | 0 | 0 | 0 | 1 | 1 | 1 | 1 | 7 |
| Onerup et al.2021 | 1 | 1 | 0 | 1 | 0 | 0 | 0 | 1 | 1 | 1 | 1 | 7 |
| Minnella et al.2019 | 1 | 1 | 1 | 1 | 0 | 0 | 1 | 1 | 1 | 1 | 1 | 8 |
| Bausys et al.2023 | 1 | 1 | 1 | 1 | 1 | 0 | 1 | 1 | 1 | 1 | 1 | 9 |
| Moug et al.2019 | 1 | 1 | 0 | 1 | 1 | 1 | 1 | 1 | 0 | 1 | 1 | 8 |
| Danielsson et al.2025 | 1 | 1 | 1 | 1 | 0 | 0 | 0 | 0 | 0 | 1 | 1 | 6 |


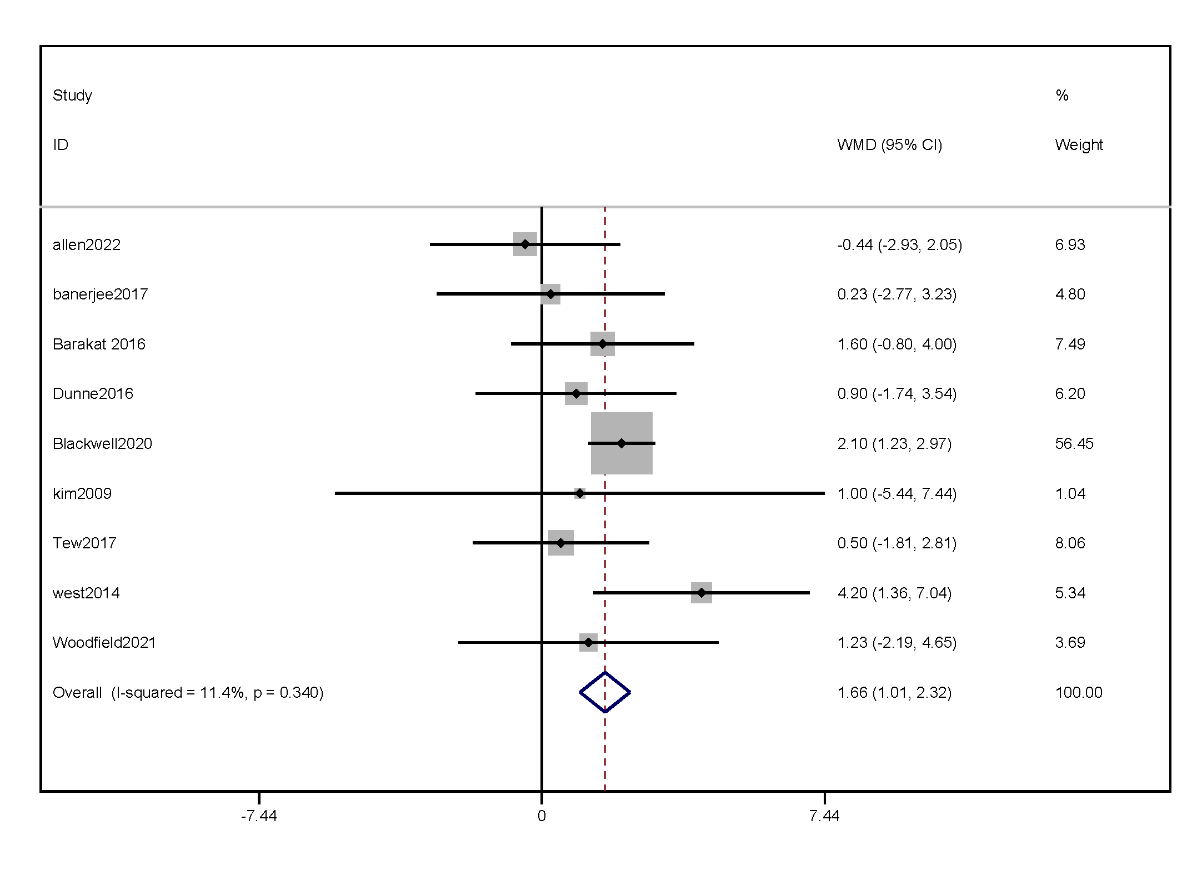


**Figure 1. Forest plot of pairwise meta-analysis for VO_2peak_.**

**Table 3 The VO_2peak_ and 6MWT rankings for different prehabilitation programs**

| Program | VO_2peak_ | | | 6MWT | | |
| --- | --- | --- | --- | --- | --- | --- |
|  | SUCRA (%) | Mean rank | P (%) | SUCRA (%) | Mean rank | P (%) |
| HIIT | 73.9 | 2.6 | 16.6 | 84.3 | 2.1 | 45.8 |
| Aerobic exercise | 62.1 | 3.3 | 45.2 | 72.9 | 2.9 | 12.7 |
| Active control | 56 | 3.6 | 13.2 | 28.4 | 6.0 | 0.4 |
| Mixed exercise | 52.9 | 3.8 | 15.2 | 37.6 | 5.4 | 0.7 |
| Physical recommendation | 50.6 | 4.0 | 2.6 | 83.9 | 2.1 | 33.9 |
| Multimodal intervention | 40.5 | 4.6 | 7.1 | 59.4 | 3.8 | 6.6 |
| ERAS protocol | - | - | - | 16.9 | 6.8 | 0 |
| Passive control | 14.0 | 6.2 | 0 | 16.5 | 6.8 | 0 |

Notes: Higher SUCRA and lower mean ranks indicate better-performing treatments. P indicates the probability of it being the best treatment.

**Table 4 Summary of GRADE assessment for the certainty in VO_2peak_**

| Comparisons | Nature of evidence | Certainty | Reason for downgrading |
| --- | --- | --- | --- |
| HIIT vs. passive control | Direct | Moderate | Imprecision |
| HIIT vs. active control | Direct | Low | Imprecision, risk of bias |
| HIIT vs. physical recommendation | Direct | Moderate | Risk of bias |
| HIIT vs. mixed exercise | Indirect | Low | Imprecision, indirectness |
| HIIT vs. multimodal intervention | Indirect | Low | Indirectness, imprecision |
| HIIT vs. aerobic exercise | Indirect | Low | Indirectness, risk of bias |
| Active control vs. passive control | Indirect | Moderate | Indirectness |
| Active control vs. physical recommendation | Indirect | Low | Indirectness, inconsistency |
| Active control vs. multimodal intervention | Indirect | Moderate | Indirectness |
| Active control vs. aerobic exercise | Indirect | Moderate | Indirectness |
| Active control vs. mixed exercise | Indirect | Low | Indirectness, risk of bias |
| Physical recommendation vs. passive control | Indirect | Low | Indirectness, imprecision |
| Physical recommendation vs. multimodal intervention | Direct | Moderate | Risk of bias |
| Physical recommendation vs. aerobic exercise | Direct | Low | Imprecision, inconsistency |
| Physical recommendation vs. mixed exercise | Indirect | Very low | Imprecision, inconsistency, indirectness |
| Multimodal intervention vs. passive control | Indirect | Low | Imprecision, indirectness |
| Multimodal intervention vs. active control | Indirect | Low | Imprecision, indirectness |
| Multimodal intervention vs. aerobic exercise | Indirect | Very low | Imprecision, inconsistency, indirectness |
| Multimodal intervention vs. mixed exercise | Indirect | Very low | Imprecision, inconsistency, indirectness |
| Mixed exercise vs. passive control | Direct | Moderate | Imprecision |
| Mixed exercise vs. active control | Indirect | Moderate | Imprecision |
| Mixed exercise vs. aerobic exercise | Indirect | Very low | Imprecision, inconsistency, indirectness |


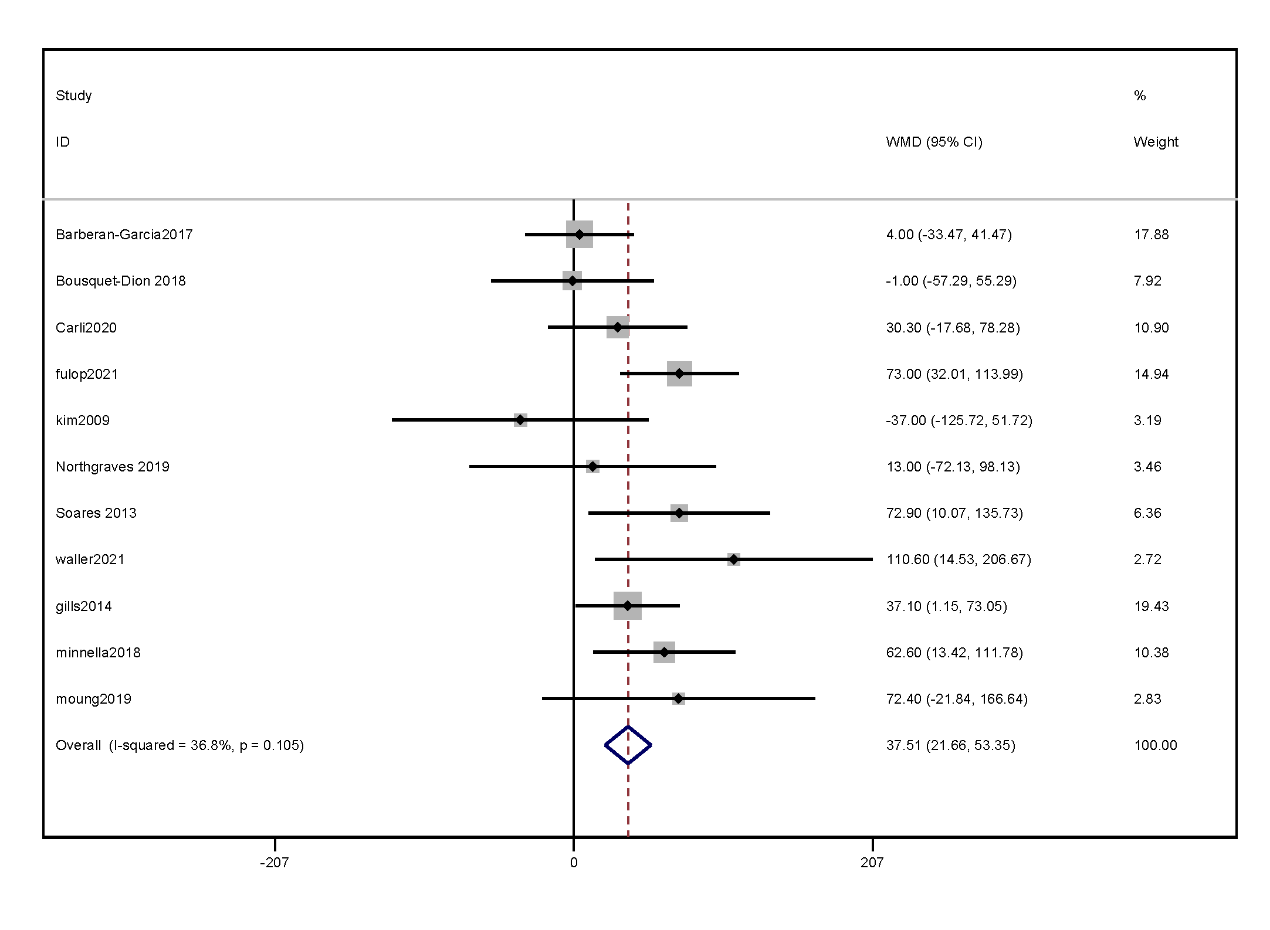


**Figure 2. Forest plot of pairwise meta-analysis for 6MWT.**


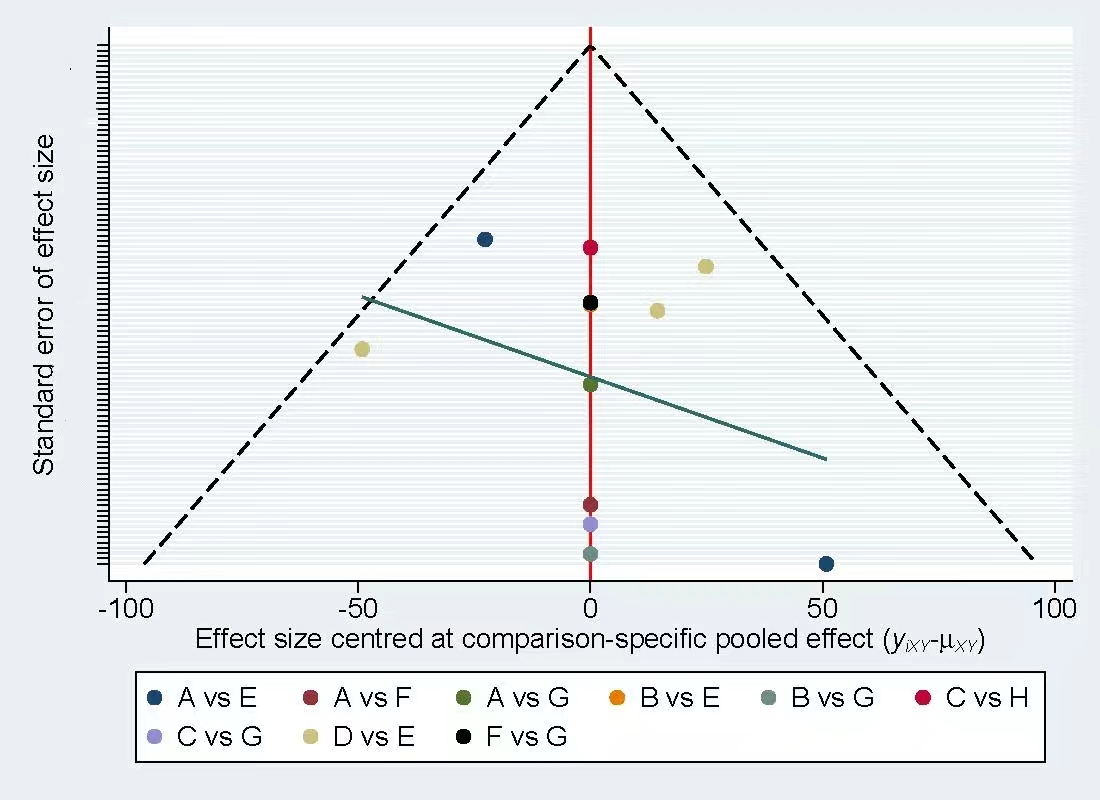


**Figure 3. Comparison-adjusted funnel plot for the 6MWT network.**

**Table 5 Summary of GRADE assessment for the certainty in 6MWT**

| Comparisons | Nature of evidence | Certainty | Reason for downgrading |
| --- | --- | --- | --- |
| HIIT vs. passive control | Indirect | Very low | Imprecision, risk of bias, indirectness |
| HIIT vs. active control | Indirect | Very low | Imprecision, risk of bias, indirectness |
| HIIT vs. physical recommendation | Mixed | Low | Imprecision, risk of bias |
| HIIT vs. mixed exercise | Indirect | Low | Imprecision, indirectness |
| HIIT vs. multimodal intervention | Indirect | Very low | Imprecision, risk of bias, indirectness |
| HIIT vs. aerobic exercise | Indirect | Very low | Imprecision, risk of bias, indirectness |
| HIIT vs. ERAS protocol | Indirect | Very low | Imprecision, risk of bias, indirectness |
| Active control vs. passive control | Indirect | Low | Imprecision, indirectness |
| Active control vs. physical recommendation | Indirect | Very low | Imprecision, risk of bias, indirectness |
| Active control vs. multimodal intervention | Mixed | Low | Imprecision, risk of bias |
| Active control vs. aerobic exercise | Mixed | Low | Imprecision, risk of bias |
| Active control vs. mixed exercise | Indirect | Low | Imprecision, indirectness |
| Active control vs. ERAS protocol | Indirect | Low | Imprecision, indirectness |
| Physical recommendation vs. passive control | Indirect | Very low | Imprecision, risk of bias, indirectness |
| Physical recommendation vs. multimodal intervention | Indirect | Low | Imprecision, indirectness |
| Physical recommendation vs. aerobic exercise | Mixed | Low | Imprecision, indirectness, indirectness |
| Physical recommendation vs. mixed exercise | Indirect | Very low | Imprecision, inconsistency |
| Physical recommendation vs. ERAS protocol | Indirect | Low | Imprecision, indirectness |
| Multimodal intervention vs. passive control | Mixed | Moderate | Imprecision |
| Multimodal intervention vs. aerobic exercise | Indirect | Very low | Imprecision, inconsistency, indirectness |
| Multimodal intervention vs. mixed exercise | Indirect | Very low | Imprecision, inconsistency, indirectness |
| Multimodal intervention vs. ERAS protocol | Mixed | Moderate | Imprecision |
| Mixed exercise vs. passive control | Mixed | Moderate | Imprecision |
| Mixed exercise vs. aerobic exercise | Mixed | Low | Imprecision, inconsistency |
| Mixed exercise vs. ERAS protocol | Indirect | Low | Imprecision, indirectness |
| ERAS protocol vs. passive control | Indirect | Low | Imprecision, indirectness |

A


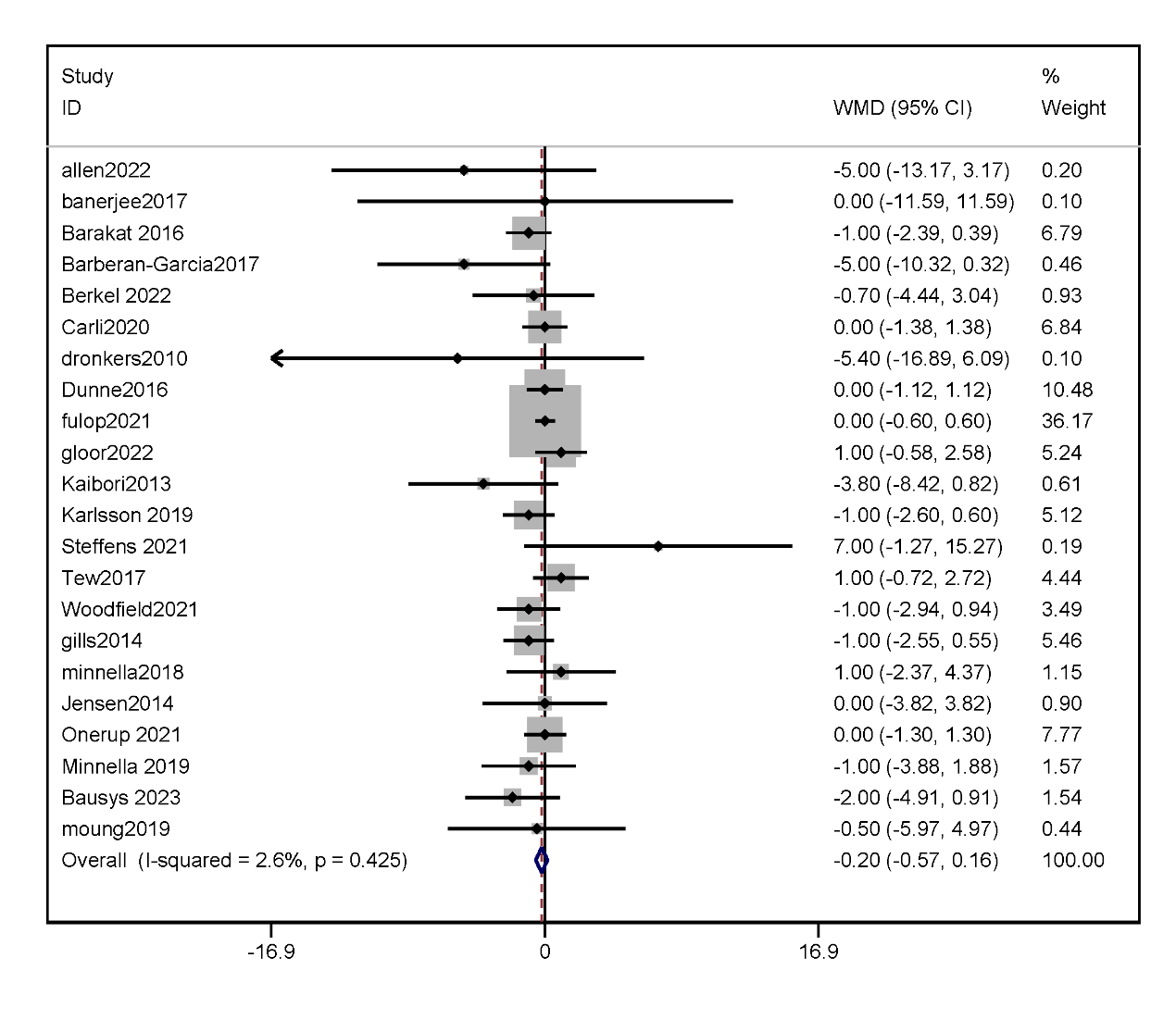


B


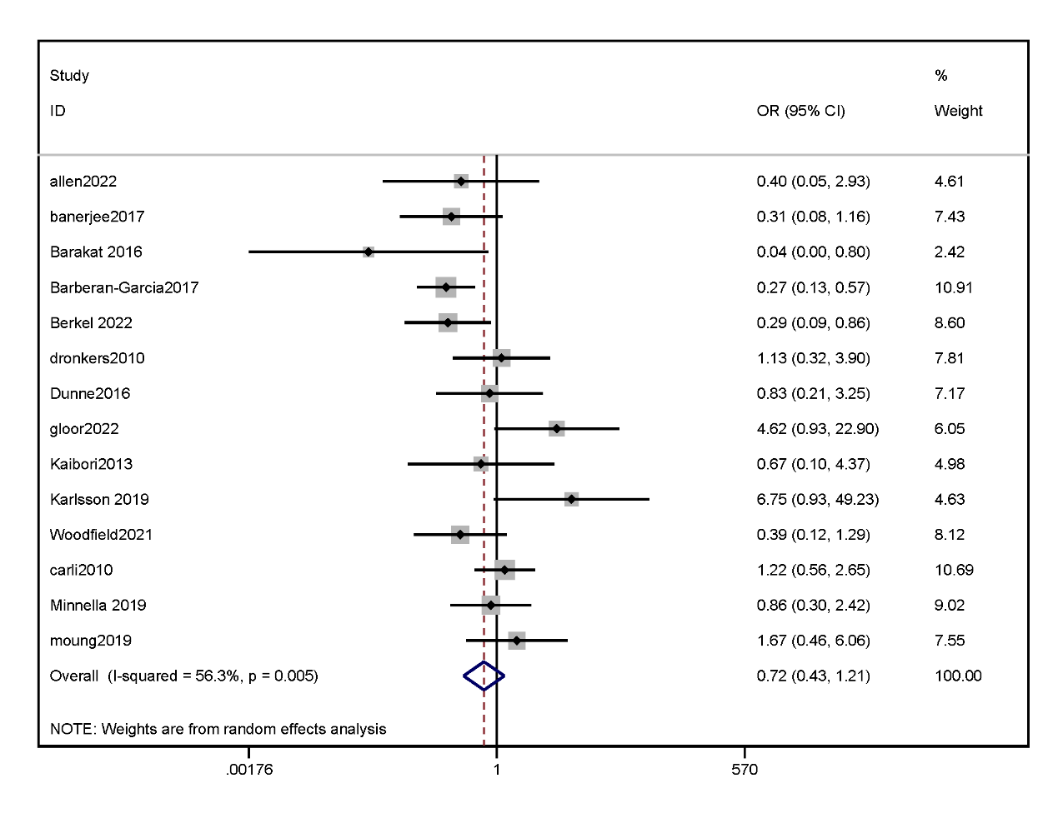


**Figure 4. (A) Forest plot of pairwise meta-analysis for LOS; (B) Forest plot of pairwise meta-analysis for post-operative complications.**

**A**

**
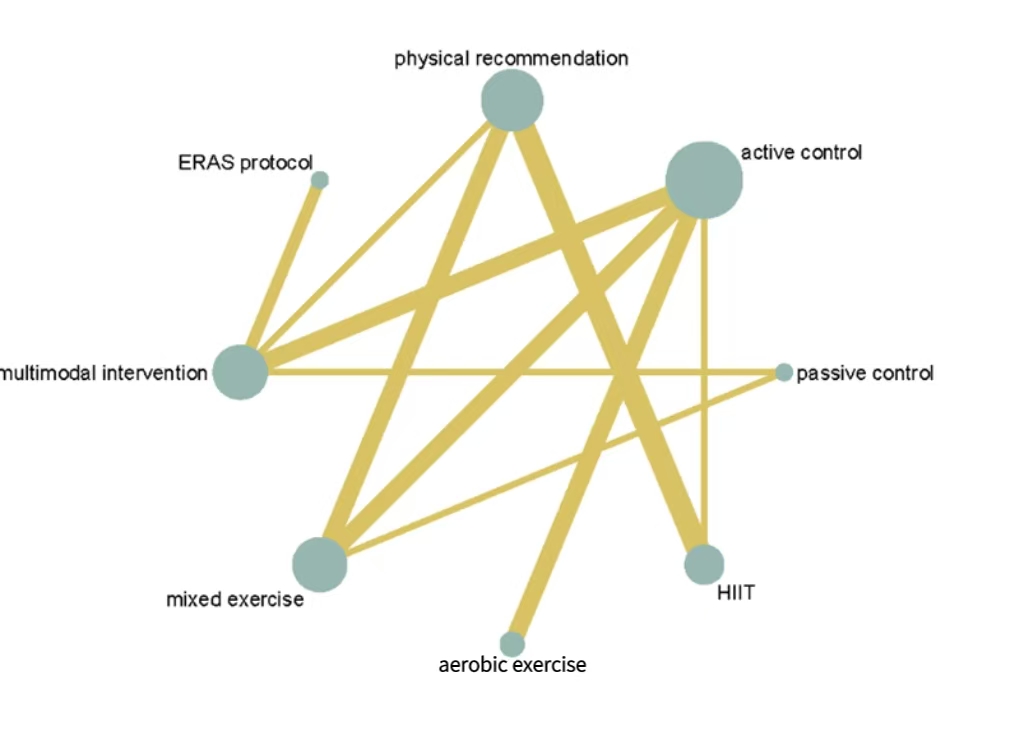
**

**B**


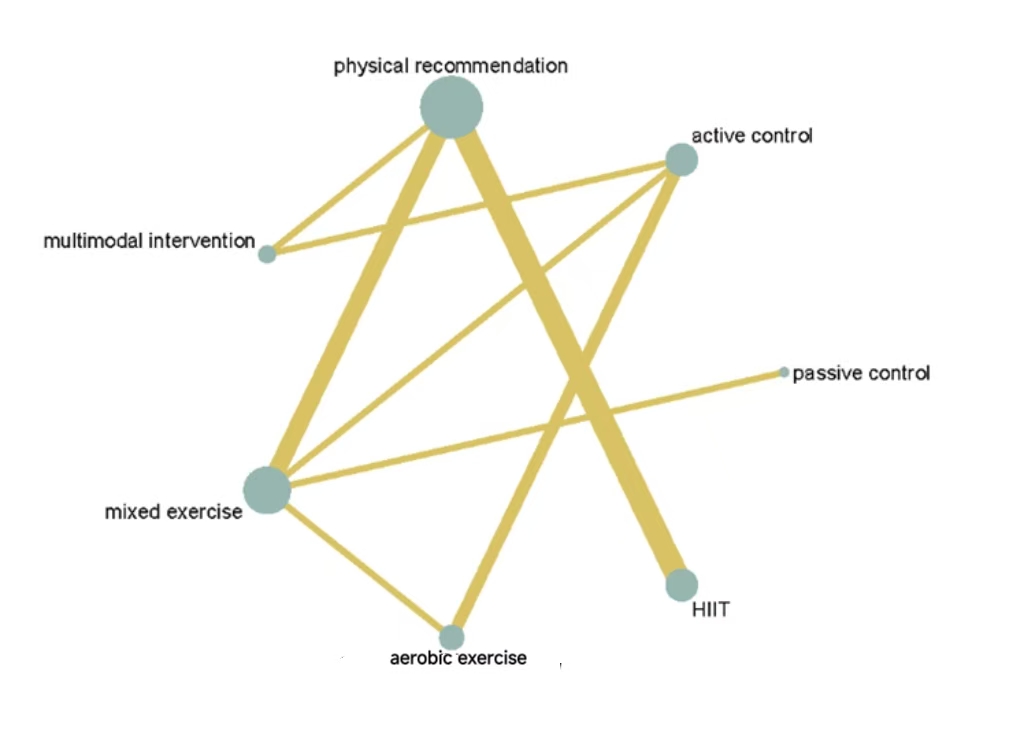


**Figure 5. Network meta-analysis of eligible comparisons for (A) LOS, (B) post-operative complications. Each node represents an intervention, and the connecting lines between 2 nodes represents 1 or more randomized clinical trials (RCTs) in which the 2 interventions have been compared directly.**

**Table 6 The LOS and post-operative complications rankings for different types of prehabilitation programs**

| Program | LOS | | | post-operative complications | | |
| --- | --- | --- | --- | --- | --- | --- |
|  | SUCRA (%) | Mean rank | P (%) | SUCRA (%) | Mean rank | P (%) |
| HIIT | 39.4 | 5.2 | 4.3 | 98.4 | 1.1 | 92.3 |
| Aerobic exercise | 71.9 | 3.0 | 34.2 | 41.9 | 4.5 | 0.8 |
| Active control | 58.8 | 3.9 | 4.3 | 32.8 | 5.0 | 0.1 |
| Mixed exercise | 39.5 | 5.2 | 3.7 | 51.1 | 3.9 | 0.1 |
| Physical recommendation | 23.7 | 6.3 | 1.7 | 67.9 | 2.9 | 0.1 |
| Multimodal intervention | 74.7 | 2.8 | 17.0 | 56.0 | 3.6 | 6.1 |
| ERAS protocol | 75.5 | 2.7 | 34.3- | - | - | - |
| Passive control | 16.5 | 6.8 | 0.5 | 1.8 | 6.9 | 0.5 |

Notes: Higher SUCRA and lower mean ranks indicate better-performing treatments. P indicates the probability of it being the best treatment.

| ERAS  protocol |  |  |  |  |  |  |  |
| --- | --- | --- | --- | --- | --- | --- | --- |
| -0.09  (-1.16,0.98) | Multimodal intervention |  |  |  |  |  |  |
| -0.08  (-2.22,2.06) | 0.01  (-1.87,1.88) | Aerobic  exercise |  |  |  |  |  |
| -0.47  (-2.20,1.25) | -0.38  (-1.65,0.88) | -0.39  (-1.93,1.15) | Active control |  |  |  |  |
| -1.01  (-3.11,1.10) | -0.92  (-2.65,0.81) | -0.93  (-3.13,1.28) | -0.53  (-2.02,1.00) | Mixed exercise |  |  |  |
| -1.00  (-3.15,1.14) | -0.92  (-2.72,0.89) | -0.92  (-3.08,1.23) | -0.53  (-2.07,1.00) | 0.00  (-1.47,1.47) | HIIT |  |  |
| -1.36  (-3.67,0.95) | -1.27  (-3.21,0.66) | -1.28  (-3.62,1.06) | -0.89  (-2.52,0.75) | -0.35  (-1.55,0.84) | -0.36  (-1.53,0.82) | Physical recommendation |  |
| -1.58  (-3.48,0.31) | -1.50  (-3.02,-0.02) | -1.50  (-3.72,0.72) | -1.11  (-2.77,0.55) | -0.58  (-2.04,0.88) | -0.58  (-2.43,1.27) | -0.22  (-2.04,1.59) | Passive control |

**Table 7. Comparative effectiveness results for LOS. Each cell shows an MD with a 95%CI. 95%CI = 95% confidence interval.**

| HIIT |  |  |  |  |  |  |
| --- | --- | --- | --- | --- | --- | --- |
| 0.60  (0.42,0.84) | Physical recommendation |  |  |  |  |  |
| 0.53  (0.24,1.16) | 0.89  (0.44,1.80) | Multimodal intervention |  |  |  |  |
| 0.52  (0.33,0.82) | 0.88  (0.64,1.19) | 0.98  (0.50,1.96) | Mixed exercise |  |  |  |
| 0.47  (0.24,0.90) | 0.79  (0.45,1.38) | 0.89  (0.44,1.79) | 0.91  (0.56,1.45) | Aerobic  exercise |  |  |
| 0.44  (0.23,0.82) | 0.73  (0.43,1.25) | 0.83  (0.48,1.42) | 0.84  (0.53,1.34) | 0.93  (0.59,1.48) | Active control |  |
| 0.03  (0.00,0.51) | 0.05  (0.00,0.84) | 0.05  (0.00,1.01) | 0.05  (0.00,0.95) | 0.06  (0.00,1.09) | 0.06  (0.00,1.17) | Passive control |

**Table 8. Comparative effectiveness results for post-operative complications. Each cell shows an OR with a 95%CI. 95%CI = 95% confidence interval.**

A


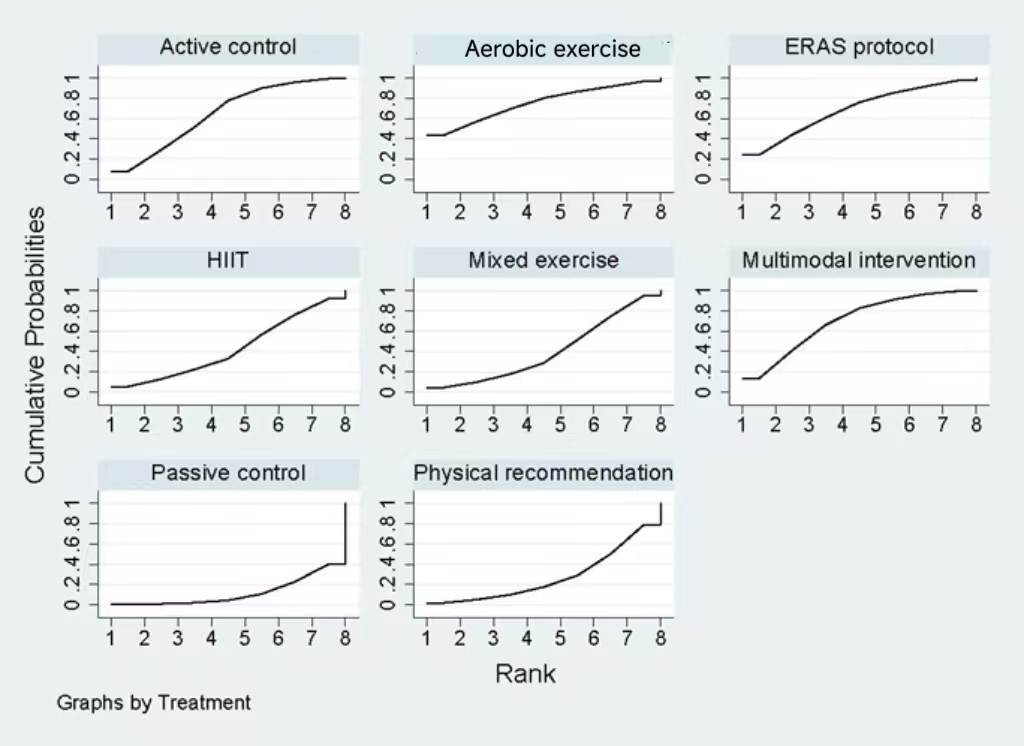


B


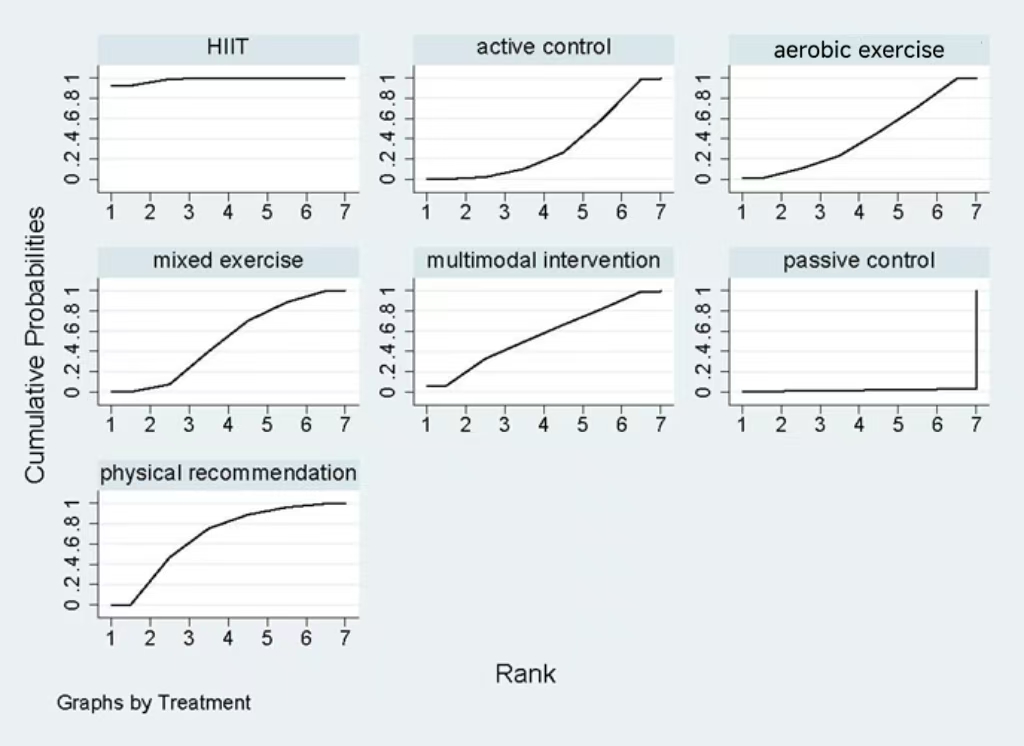


**Figure 6. Cumulative ranking probability plots for (A) LOS and (B) post-operative complications. The horizontal axis represents the possible rank of each treatment (from best to worst according to the outcome). The vertical axis represents the cumulative probability for each treatment to be the best option, the best of 2 options, the best of 3 options, and so on.**

A


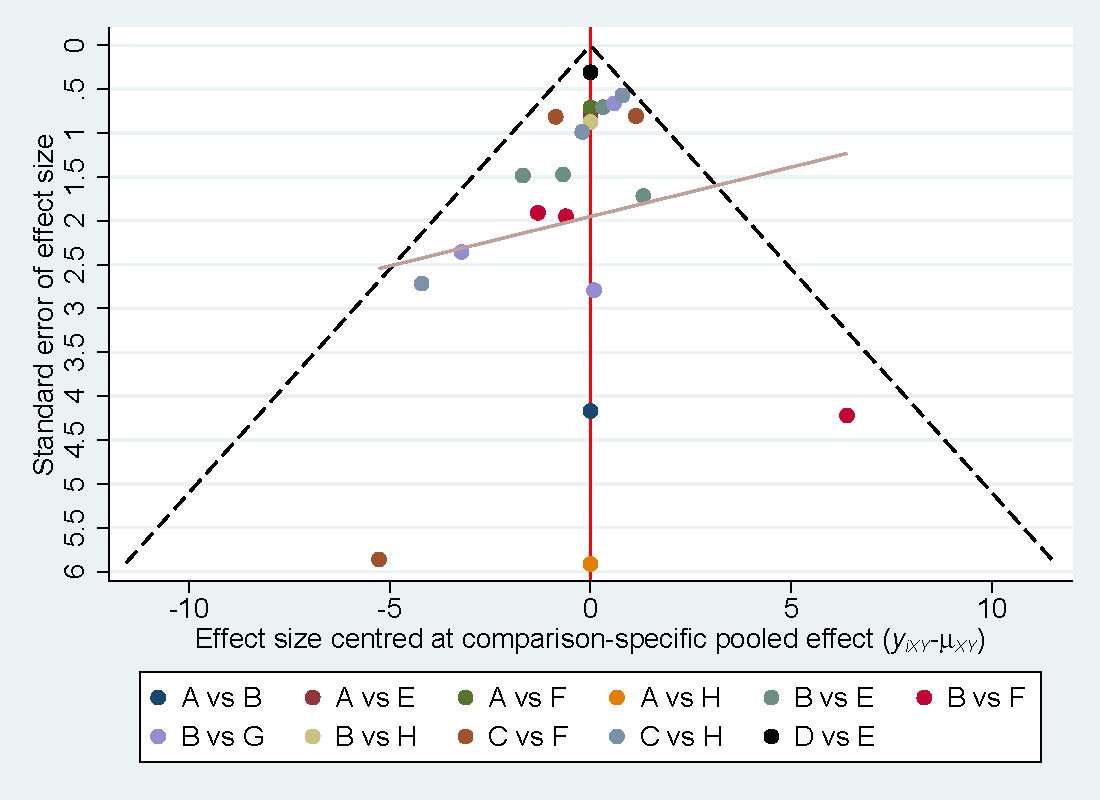


B


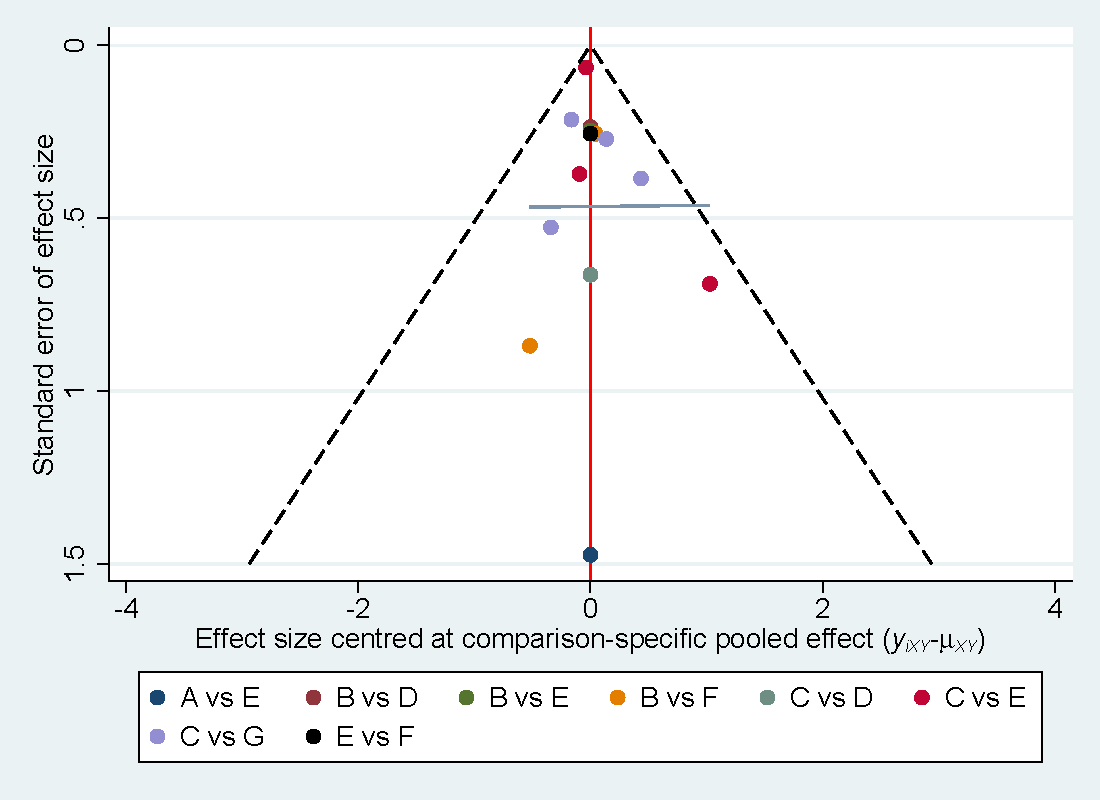


**Figure 7. (A) Comparison-adjusted funnel plot for the LOS network; (B) Comparison-adjusted funnel plot for the network post-operative complications.**

**Table 9 Summary of GRADE assessment for the certainty in LOS**

| Comparisons | Nature of evidence | Certainty | Reason for downgrading |
| --- | --- | --- | --- |
| HIIT vs. passive control | Indirect | Moderate | Indirectness |
| HIIT vs. active control | Mixed | High | No downgrade |
| HIIT vs. physical recommendation | Mixed | Moderate | Risk of bias |
| HIIT vs. mixed exercise | Indirect | Low | Inconsistency, indirectness |
| HIIT vs. multimodal intervention | Indirect | Low | Inconsistency, indirectness |
| HIIT vs. aerobic exercise | Indirect | Very low | Inconsistency, indirectness Imprecision |
| HIIT vs. ERAS protocol | Indirect | Low | Imprecision, indirectness |
| Active control vs. passive control | Indirect | Low | Imprecision, indirectness |
| Active control vs. physical recommendation | Indirect | Low | Imprecision, indirectness |
| Active control vs. multimodal intervention | Mixed | Moderate | Imprecision |
| Active control vs. aerobic exercise | Mixed | Moderate | Imprecision |
| Active control vs. mixed exercise | Mixed | Moderate | Imprecision |
| Active control vs. ERAS protocol | Indirect | Low | Risk of bias, indirectness |
| Physical recommendation vs. passive control | Indirect | Very low | Risk of bias, indirectness, imprecision |
| Physical recommendation vs. multimodal intervention | Mixed | Moderate | Imprecision |
| Physical recommendation vs. aerobic exercise | Indirect | Low | Imprecision, indirectness |
| Physical recommendation vs. mixed exercise | Mixed | Moderate | inconsistency |
| Physical recommendation vs. ERAS protocol | Indirect | Low | Imprecision, indirectness |
| Multimodal intervention vs. passive control | Mixed | Moderate | Imprecision |
| Multimodal intervention vs. aerobic exercise | Indirect | Low | Imprecision, indirectness |
| Multimodal intervention vs. mixed exercise | Indirect | Very low | Imprecision, inconsistency, indirectness |
| Multimodal intervention vs. ERAS protocol | Direct | Moderate | Imprecision |
| Mixed exercise vs. passive control | Mixed | Moderate | Imprecision |
| Mixed exercise vs. aerobic exercise | Indirect | Very low | Imprecision, inconsistency, indirectness |
| Mixed exercise vs. ERAS protocol | Indirect | Low | Imprecision, indirectness |
| ERAS protocol vs. passive control | Indirect | Low | Imprecision, indirectness |

Table 10 Summary of GRADE assessment for the certainty in post-operative complications

| Comparisons | Nature of evidence | Certainty | Reason for downgrading |
| --- | --- | --- | --- |
| HIIT vs. passive control | Indirect | Low | Indirectness, imprecision |
| HIIT vs. active control | Indirect | Moderate | Indirectness |
| HIIT vs. physical recommendation | Direct | High | No downgrade |
| HIIT vs. mixed exercise | Indirect | Low | Inconsistency, indirectness |
| HIIT vs. multimodal intervention | Indirect | Low | Inconsistency, indirectness |
| HIIT vs. aerobic exercise | Indirect | Low | Inconsistency, indirectness |
| Active control vs. passive control | Indirect | Low | Imprecision, indirectness |
| Active control vs. physical recommendation | Indirect | Low | Inconsistency, indirectness |
| Active control vs. multimodal intervention | Mixed | Moderate | Imprecision |
| Active control vs. aerobic exercise | Mixed | Moderate | Imprecision |
| Active control vs. mixed exercise | Mixed | Moderate | Imprecision |
| Physical recommendation vs. passive control | Indirect | Very low | Inconsistency, indirectness, Imprecision |
| Physical recommendation vs. multimodal intervention | Mixed | Moderate | Risk of bias |
| Physical recommendation vs. aerobic exercise | Indirect | Very low | Inconsistency, indirectness, risk of bias |
| Physical recommendation vs. mixed exercise | Mixed | Moderate | Inconsistency |
| Multimodal intervention vs. passive control | Indirect | Low | Imprecision, indirectness |
| Multimodal intervention vs. aerobic exercise | Indirect | Low | Inconsistency, indirectness |
| Multimodal intervention vs. mixed exercise | Indirect | Low | Risk of bias, indirectness |
| Mixed exercise vs. passive control | Direct | Moderate | Imprecision |
| Mixed exercise vs. aerobic exercise | Mixed | Moderate | Inconsistency |

**Stata Code**

network setup mean sd n, study(id) trt(t) format(augment)

network map

network meta i

network meta c

network forest

network sidesplit all, tau

network rank min, all zero reps(5000) gen(prob)

sucra prob*, lab( )

netleague, lab( ) sort( ) export ("D:\network.xlsx")

intervalplot, null(0) lab( )

network convert pairs

netfunnel _y _stderr _t1 _t2 , random bycomp add(lfit _stderr _ES_CEN) noalpha

ifplot _y _stderr _t1 _t2 id, tau2(loop)

netweight _y _stderr _t1 _t2
